# Treatment resistant schizophrenia: a comprehensive survey of randomised controlled trials

**DOI:** 10.1186/s12888-014-0253-4

**Published:** 2014-09-12

**Authors:** Diarmid Sinclair, Clive E Adams

**Affiliations:** Sheffield Health and Social Care NHS Foundation Trust, Fullwood House, 5 Old Fulwood Rd, Sheffield, South Yorkshire S10 3TG UK; Cochrane Schizophrenia Group, Institute of Mental Health, University of Nottingham, Nottingham, UK

**Keywords:** Systematic reviews, Schizophrenia, Treatment resistant, Randomised trials

## Abstract

**Background:**

Schizophrenia is a common serious mental health condition which has significant morbidity and financial consequences. The mainstay of treatment has been antipsychotic medication but one third of people will have a ‘treatment resistant’ and most disabling and costly illness. The aim of this survey was to produce a broad overview of available randomised evidence for interventions for people whose schizophrenic illness has been designated ‘treatment resistant’.

**Method:**

We searched the Cochrane Schizophrenia Group’s comprehensive Trials Register, selected all relevant randomised trials and, taking care not to double count, extracted the number of people randomised within each study. Finally we sought relevant reviews on the Cochrane Library and investigated how data on this subgroup of people had been presented.

**Results:**

We identified 542 relevant papers based on 268 trials (Average size 64.8 SD 61.6, range 7–526, median 56 IQR 47.3, mode 60). The most studied intervention is clozapine with 82 studies (total n = 6299) comparing it against other anti-psychotic medications. Cognitive behavioural therapy (CBT), electroconvulsive therapy (ECT), or transcranial magnetic stimulation (TMS) supplementing a standard care and risperidone supplementation of clozapine has also been extensively evaluated within trials. Many approaches, however, were clearly under researched. There were only four studies investigating combinations of non-clozapine antipsychotics. Only two psychological approaches (CBT and Family Rehabilitation Training) had more than two studies. Cochrane reviews rarely presented data specific to this important clinical sub-group.

**Conclusions:**

This survey provides a broad taxonomy of how much evaluative research has been carried out investigating interventions for people with treatment resistant schizophrenia. Over 280 trials have been undertaken but, with a few exceptions, most treatment approaches - and some in common use - have only one or two relevant but small trials. Too infrequently the leading reviews fail to highlight the paucity of evidence in this area – as these reviews are maintained this shortcoming should be addressed.

**Electronic supplementary material:**

The online version of this article (doi:10.1186/s12888-014-0253-4) contains supplementary material, which is available to authorized users.

## Background

Schizophrenia is a common serious mental health condition affecting approximately 1% of the population [1]. The course of illness is variable with a minority fully recovering from an initial episode whilst most with have a relapsing remitting course [2]. Antipsychotic medication has been part of the standard care of schizophrenia since the introduction of chlorpromazine in 1952. Up to 60% of people with schizophrenia will respond to antipsychotic medication but about 1 in 3 people have an illness that is “treatment resistant” [3] and this group suffer, cause problems to their carers and society, and are enormously resource-intensive – often for decades [4].

Treatment resistant schizophrenia (TRS) has not been consistency defined within the literature [5]. In a landmark randomised trial John Kane and colleagues investigated the effects of clozapine compared with chlorpromazine for people with TRS [6]. In this study TRS was defined as “at least three periods of treatment with antipsychotics from at least two different classes at adequate doses for an adequate period time with no relief and no period of good functioning over the last five years”. Kane’s trial led to clozapine’s reintroduction to common use and the criteria used to define TRS in this study remain some of the most cited in randomised trials concerning TRS [7].

Clozapine's use is restricted partly due to serious adverse effects including blood disorders and cardiac toxicity. Many newer agents or approaches have been the focus of TRS studies with the hope of having similar efficacy to clozapine but better tolerability and safety – although, on average, other medications seem less effective than clozapine [8,9]. Whilst clozapine is the only licensed drug for TRS, clinicians do often try other approaches such as poly-pharmacy and high dose prescribing [10] before its prescription with an average 47.7 months delay in use of clozapine [11]. Up to 30% of people whose illness has been designated as resistant to treatment will have an inadequate response to clozapine and more interventions may be indicated [12]. Interventions in TRS include not only pharmacological interventions but also psychological and ‘physical’ interventions such as electroconvulsive therapy (ECT). This important subgroup of people is an active area of evaluative research and we know of no overview and taxonomy.

Small trials can lead to imprecise results where there are wide 95% confidence intervals (CIs). Wide CIs can lead to difficulties in making clinical recommendations especially where these cross the line of no effect suggesting a treatment is potentially ineffective. As study size increases so does precision of results leading to a more accurate estimate of the effect size. It has also been shown that trials containing small numbers of randomised participants can lead to treatment effects being overestimated and that these can decrease as more data becomes available [13]. This may be due to a variety of factors such as low methodological quality but may also be due to a failure of the randomisation to balance differences between control and intervention groups in terms of prognosis due to random error. The GRADE recommendations take account of this and suggest rating down recommendations based on imprecise results or studies containing small numbers of participants [14].

### Aim

To produce a broad overview of available randomised evidence (pharmacological or non-pharmacological) for interventions in TRS and identify areas where further research and data synthesis may be beneficial.

## Methods

The Cochrane Schizophrenia Group (CSG) register was searched in October 2012. This register is compiled by methodical searches of 70 different biomedical databases including BIOSIS, CINAHL, Dissertation abstracts, EMBASE, LILACS, MEDLINE, PSYNDEX, PsycINFO, RUSSMED, Sociofile and is supplemented with hand searching of relevant journals and numerous conference proceedings. This strategy attempts to reduce the risk of publication bias. A detailed account of the group’s search strategy is available [15].

The search terms used were: *Treatment?resist*, *non?respon*, *non?remission*, *non?remitter*, *Therapy?Resist*, *treatment?refract*, *medication?resistan*, *drug?resistan*.

All abstracts returned by this search were then inspected for relevance. If there was any doubt from the abstract alone, the full paper was obtained for clarification. Studies not concerning therapeutic interventions, not mentioning treatment resistant schizophrenia, or that were not randomised were excluded. Given the lack of consensus criteria for diagnosing TRS a broad definition was adopted whereby a paper was included if the study population was deemed to be treatment resistant by the authors. We also included papers where the authors did not explicitly mention treatment resistance but there was evidence that all participants had tried pharmacological interventions but these had not been effective or the patients were intolerant of them prior to the study intervention. There was no requirement for the papers to report any particular outcome measure or length of follow-up to be included. Multiple reports of single trials were grouped to avoid double counting. A single trial may appear in numerous publications and, if not corrected for, could introduce spurious precision by being counted over and over again. Trials were then grouped by type of intervention and data extracted on number of participants. Data were extracted onto an electronic database. The data extraction was undertaken independently by DS. The primary outcome of interest was the number of participants in a study so to produce an indication how much RCT evidence there was for any particular intervention. Relevant Cochrane reviews were identified and inspected.

## Results

**Figure 1:**
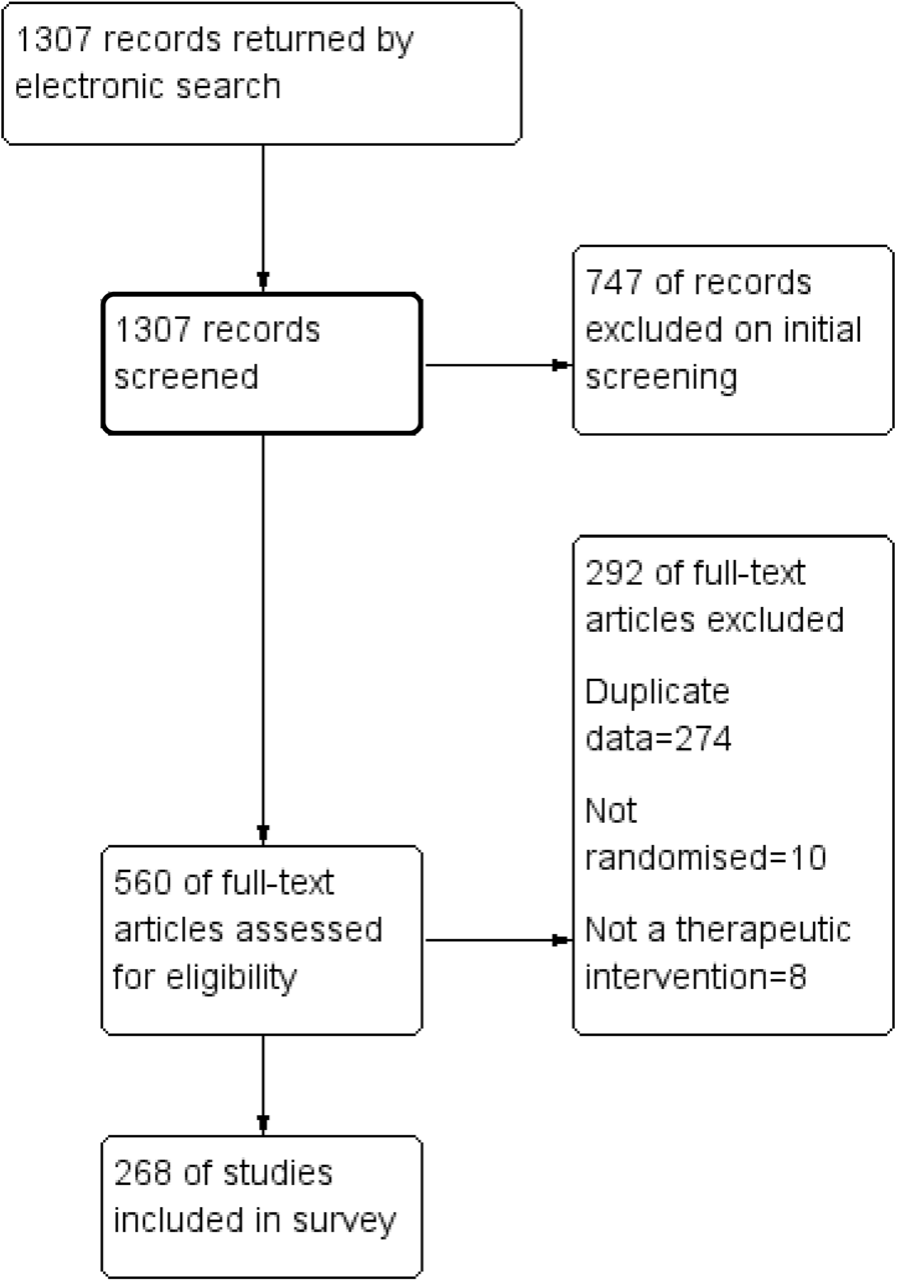
**Literature search flow diagram.**

The initial electronic search returned 1307 references (Figure 1). 560 of these were relevant to this review. After excluding duplicates, non-randomised studies and studies not concerning a therapeutic intervention there were 268 included studies. Relevant interventions tested in randomised trials fell into four broad categories (Tables 1, 2, 3 and 4). The average size of trials was 64.8 (SD 61.6, range 7–526, median 56, mode 60) and the most commonly evaluated treatments are cognitive behavioural therapy, electroconvulsive therapy, or transcranial magnetic stimulation supplementing a standard care which would include antipsychotic treatment, risperidone supplementation of clozapine, or a list of antipsychotics (most notably clozapine) as stand-alone treatment for people whose illness had been designated resistant to treatment (Table 4). Cochrane reviews, not specifically focusing on clozapine, provide extensive coverage of the data from within broad groups of trials, but few of these leading reviews provide specific consideration of this important sub-group of people (Additional file 1: Table S1).

**Table 1:** **Non-pharmacological interventions – added to standard care**

**Type of intervention**	**Number**		Cochrane review*
	**Studies**	**Participants**	
**Psychological**			
Attention shaping	1	82	
Cognitive behavioural therapy	13	824	[16]
Cognitive behavioural therapy +	1	38	
**D-Cycloserine**			
Emotion management training	1	22	
Family behavioural therapy	1	30	[17]
Family rehabilitation training	2	91	[17]
Integrative therapy	1	76	
Neuropsychological rehabilitation therapy	1	93	
Occupational therapy	1	26	
**Alternative medicine**			
Acupuncture	1	40	[18]
**Physical treatments**			
Electroconvulsive therapy	13	886	[19]
Transcranial magnetic stimulation	15	423	[20]*
Psychosurgery	1	36	
Transcranial direct-current stimulation	2	120	
Intravascular irradiation	1	60	
Haemodialysis	1	11	

*Protocols.

**Table 2:** **Adjuvant interventions – added to clozapine**

**Name of intervention**	**Number**		**Cochrane review**
	**Studies**	**Participants**	
**Antipsychotics**			
**Amisulpride**	4	322	[21]
**Aripiprazole**	5	349	
**Fluphenazine**	1	60	
**Haloperidol**	2	116	
**Paliperidone**	1	70	
**Pipotiazine**	1	84	
**Quetiapine**	2	136	[21]
**Risperidone**	10	646	[21]
**Sertindole**	1	50	
**Sulpride**	2	150	[21,22]
**Ziprasidone**	3	150	[21]
**Clozapine added to ‘other antipsychotics’**	2	100	
**Antidepressants**			
**Duloxetine**	1	33	
**Fluvoxamine**	1	68	
**Anticonvulsants/mood stabilisers**			
**Valproate**	1	34	[23]*
**Lamotrigine**	2	85	[24]*
**Lithium**	3	152	[25]*
**Antimicrobials**			
**Inosine**	1	66	
**Herbal**			
**Ginkgo Biloba**	1	42	
**Anti-diabetic**			
**Metformin**	1	61	
**Selective norepinephrine reuptake inhibitor**			
**Atomoxetine**	1	126	
**Drugs Used in Dementia**			
**Mematine**	1	21	[26]**
**Amino acids**			
**Glycine**	2	31	[27]*

*Review includes interventions that weren’t just added to clozapine.

**Protocol and intervention wasn’t specifically added to clozapine.

**Table 3:** **Adjuvant interventions – added to antipsychotics other than clozapine**

**Name of intervention**		**Number**		**Cochrane review**
		**Studies**	**Participants**	
**Added to specific antipsychotic**				
**Olanzapine**				
Antipsychotic	Chlorpromazine	1	39	
	Sulpride	2	114	
**Risperidone**				
Antipsychotic	Quetiapine	1	144	
**Chlorpromazine**				
Movement disorder drugs	LDOPA	1	8	
**Haloperidol**				
Serotonin 5-HT_3_ receptor antagonist	Ondansetron	1	121	
**Added to unspecified antipsychotic**				
Amino Acids	Glycine	3	50	[27]
	Serine	1	39	[27]
Antidepressants	Citalopram	1	18	
	Escitalopram	1	30	
	Mianserin	1	18	
	Sertraline	1	77	
Anticonvulsants and mood stabilisers	Valproate	2	140	[23]
	Carbamazepine	3	79	[28]
	Topiramate	2	86	
	Lamotrigine	1	38	[24]
	Lithium	5	227	[25]
Antimicrobials	D-Cycloserine	2	35	[27]
	Ketoconazole	2	24	
Herbal	Ning Xin Tang	1	60	
	Ginkgo Biloba	1	82	
	Yi Gan San	1	59	
Movement disorder drugs	Apomorphine	1	18	
	Tetrabenazine	1	41	
Opioid acting drugs	Methadone	1	7	
Salt or ester of benzoic acid	Benzoate	1	60	
Histamine H_2_ receptor antagonist	Famotidine	1	30	
Oestrogen receptor antagonist	Tamoxifem	1	26

**Table 4:** **Non-adjuvant use of antipsychotic medication**

	**Number**		**Cochrane review**
**Name of experimental intervention**	**Studies**	**Participants**	
**Amisulpride**			
vs Antipsychotics which include clozapine	1	140	[29]
**Aripiprazole**			
vs Antipsychotics which include clozapine	8	591	[30]
vs Antipsychotics excluding clozapine	4	511	[30]
**Clozapine**			
Dosing levels	5	236	
vs Non-clozapine antipsychotics	82	6299	[31,32]
**Olanzapine**			
vs Antipsychotics which include clozapine	19	1381	[33]
vs Antipsychotics excluding clozapine	8	899	[33]
**Risperidone**			
Dosing levels	1	27	[34]
vs Antipsychotics which include clozapine	32	2380	[35]
vs Antipsychotics excluding clozapine	14	1108	[36]
**Quetiapine**			
Dosing levels	1	60	
vs Antipsychotics which include clozapine	7	615	[37]
vs Antipsychotics excluding clozapine	4	503	[37]
**Sertindole**			
vs Antipsychotics excluding clozapine	1	321	[38]
**Ziprasidone**			
vs Antipsychotics which include clozapine	4	445	[39]
vs Antipsychotics excluding clozapine	1	306	[39]
**Chlorpromazine**			
vs Antipsychotics which include clozapine	4	525	[40]**
vs Antipsychotics excluding clozapine	10	1019	[40]**
**Fluphenazine**			
Dosing levels	1	31	
vs Antipsychotics which include clozapine	1	21	[41]**
vs Antipsychotics excluding clozapine	4	185	[42]**
**Haloperidol**			
Dosing levels	1	11	
vs Antipsychotics which include clozapine	8	983	
vs Antipsychotics excluding clozapine	15	1294	[43]**
**Perphenazine**			
vs Antipsychotics excluding clozapine	1	300	[44]**
**Thiothexene**			
Dosing levels	2	82	
Others* (Interventions with less than 50 participants)			
Dosing levels	2	143	
vs Antipsychotics excluding clozapine	4	94	

*Flupentixol, Levopromazine, Lurasidone, Mesoridazine, Paliperidone, Remoxipride, Thioridazine.

**Protocol.

## Discussion

The James Lind Alliance has recently focused on gaining a UK consensus for prioritisation of research topics in schizophrenia for clinicians, researchers and patients. This broad process results in a top ten topic list and top of that list was “What is the best way to treat people with schizophrenia that is unresponsive to treatment?” [45]. Whilst we have not assessed the quality of the research this survey does give a broad taxonomy of how much evaluative research has been carried out investigating interventions for people with treatment resistant schizophrenia. We are aware of discrepancies between the numbers we present and the study count in some key systematic reviews – almost invariably with the paper published review containing considerably less studies than we have listed in the tables (for example [46]). This may be a function of the reviewers selecting studies of highest quality, and issues of currency and comprehensiveness of searches.

It is good to see how evaluative effort has some focuses – such on the evolution of adjunctive cognitive behavioural therapy, or use of clozapine – but it still seems that there are considerable efforts made by pioneering and entrepreneurial researchers and funders on trials focusing on any number of treatments. Common approaches to treatment are under-researched. For example, poly-pharmacy is prevalent and may be used in preference to initiating clozapine in people whose illness is resistant to treatment [47]. There are, however, only four studies investigating combinations of non-clozapine antipsychotics (total of 297 participants). Also, evidence for augmenting antipsychotics with anti-depressant medication seems lacking as no individual drug had more than one study and these trials included only 244 participants in total.

The great majority of trials right across this area seem grossly underpowered to find any clinically important outcome. Understandably, much evidence synthesis has been undertaken but key *maintained* reviews (Cochrane reviews) have largely been too broad to provide synthesis of evidence for this important sub-group of people. When that is not the case, and synthesis of all trials relevant to those with treatment resistant illness is undertaken and presented, these reviews report salutary lessons on the limited clinical conclusions to be drawn from even the totality of evidence [16-44].

### Limitations

This survey has not examined the effect sizes of any of the interventions involved. Some interventions may have only a low number of relatively small RCTs but if the intervention were to have a large effect size then there could be robust evidence already for the intervention being beneficial or harmful, although there is some evidence that such findings may be misleading due to the imprecision of small studies [13].

It was beyond the scope of this survey to examine the methodology of all the included studies in detail and as a result some studies that are included may be of low quality with serious risk of bias. This would mean that some areas may look like they have a robust evidence base when this is not the case.

There is also no universally accepted definition of what constitutes TRS. This survey had a very broad definition of treatment resistance meaning that for a narrower definition there would be less included studies. As a result there is likely to be heterogeneity between studies.

## Conclusions

The thousands of people with treatment resistant schizophrenia who have invested time, effort and trust may be heartened to know that some direction has emerged from the not quite so random activity. The management of treatment resistant schizophrenia does remain controversial [48] but guidelines have some consistency in supporting use of clozapine [49,50] and psychosocial approaches for which service users have helped generate the greatest body of evidence. Participants in trials may be far less pleased to see how often they have been persuaded to give informed consent to trials that are never likely to really provide clinically important outcomes.

Too often funders and researchers do not seem to communicate or collaborate. We do not need more individual trials of 100 people unless part of a network of other studies. Such studies are likely to overestimate the effects of experimental treatments [51] and are unlikely to give a precise enough estimate of effect size and may lead to Type I or Type II errors [13,52]. Two arm randomised trials with a total of 300 participants have power to clearly show a difference of 20% between groups for binary outcomes such as ‘better’ or ‘not better’ (α 0.05, β 20%). We suggest that there are enough studies of about 100 participants to act as pilots for larger trials. Underpowered studies leave everyone depending on interpretation of scales such as BPRS [53] that are rarely used in clinical practice. Many such scales are proxy measures for real clinical outcomes. There are difficulties in determining whether changes in, for example, BPRS scores translate into clinically meaningful changes for patients with evidence suggesting higher cut-offs are required [54]. GRADE guidelines would also suggest downgrading recommendations based on small numbers of data [14] and only 33 studies contained more than 100 participants. This meant lots of participants were being enrolled onto studies that were unlikely to change clinical practice.

Systematic reviews should make data accessible on this important sub-group of people. This area of care is of concern to everyone [45] but too often good reviews leave data inaccessible. With maintenance of these reviews this shortcoming could be addressed.

## Additional file

Additional file 1: Table S1. Non-pharmacological interventions. **Table S2.** Adjuvant interventions – added to clozapine. **Table S3.** Adjuvant interventions – added to antipsychotics other than clozapine. **Table S4.** Non-adjuvant use of antipsychotic medication.
