# Supplementary material for: Treatment resistant schizophrenia: a comprehensive survey of randomised controlled trials
Source: BMC Psychiatry. 2014 Sep 12;14:253. doi: 10.1186/s12888-014-0253-4 (PMC4177431; doi:10.1186/s12888-014-0253-4)
Supplement: Additional file 1: Table S1. — Non-pharmacological interventions. Table S2. Adjuvant interventions – added to clozapine. Table S3. Adjuvant interventions – added to antipsychotics other than clozapine. Table S4. Non-adjuvant use of antipsychotic medication. [file 12888_2014_253_MOESM1_ESM.docx]

**Table 1 Non-pharmacological interventions**

| Study | Number of Patients Randomised |
| --- | --- |
| Psychological Therapies | |
| Attention Shaping | |
| Silverstein et al., 2009 | 82 |
| Total Studies=1 | **Total Randomised=82** |
| Cognitive Behavioural Therapy | |
| Birchwood et al., 2011 | 180* |
| Clarke, 2011 | 80* |
| de Paiva Barretto et al., 2009 | 22 |
| Durham et al., 2003 | 66 |
| Freeman et al., 1998 | 60 |
| Haddock et al., 1998 | 33 |
| Hayward et al., 2009 | 39 |
| Neil A Rector, 2003 | 42 |
| Penn, 2004 | 60 |
| Pinto et al., 1999 | 41 |
| Sensky et al., 2000 | 90 |
| Tarrier et al., 1993 | 49 |
| Valmaggia et al., 2005 | 62 |
| Total Studies=13 | **Total Randomised=824** |
| Cognitive Behavioural Therapy + D-cycloserine | |
| Goff et al., 2011 | 38 |
| Total Studies=1 | **Total Randomised=38** |
| Emotion Management Training | |
| Hodel et al., 2004 | 22 |
| Total Studies=1 | **Total Randomised=22** |
| Family Behaviour Therapy | |
| Zastowny et al., 1992 | 30 |
| Total Studies=1 | **Total Randomised=30** |
| Family Rehabilitation Training | |
| Zheng, 2006 | 91 |
| Total Studies=1 | **Total Randomised=91** |
| Integrative Therapy | |
| Jenner et al., 2006 | 76 |
| Total Studies=1 | **Total Randomised=76** |
| Neuropsychological rehabilitation therapy | |
| Ojeda et al., 2012 | 93 |
| Total Studies=1 | **Total Randomised=93** |
| Occupational Therapy | |
| Buchain et al., 2003 | 26 |
| Total Studies=1 | **Total Randomised=26** |
| Alternative medicine | |
| Acupuncture | |
| Xiong et al., 2010 | 40 |
| Total Studies=1 | **Total Randomised=40** |

| Study | Number of Patients Randomised |
| --- | --- |
| Physical Therapies | |
| Electroconvulsive Therapy | |
| Braga, 2009 | 38 |
| Cai, 2008 | 100 |
| Chanpattana et al., 1999 | 51 |
| Ding, 2007 | 60 |
| Gao and Zhang, 2009 | 65 |
| Goswami et al., 2003 | 25 |
| JIANG et al., 2009 | 69 |
| Neumann, 1988 | 16 |
| Oleneva, 2005 | 40 |
| Tang and Ungvari, 2003 | 30 |
| Wang, 2008 | 74 |
| 刘发强 and 龚高钦, 2010 | 72 |
| 张轶杰 et al., 2010 | 246 |
| Total Studies=13 | **Total Randomised=886** |
| Transcranial magnetic stimulation | |
| Blumberger et al., 2012 | 54 |
| Brunelin et al., 2006 | 24 |
| Daskalakis, 2007 | 30 |
| Fitzgerald et al., 2005 | 33 |
| Fitzgerald et al., 2008 | 20 |
| Hoffman RE et al., 2003 | 24 |
| Klírová et al., 2009 | 10 |
| Lee et al., 2005 | 39 |
| McIntosh et al., 2004 | 16 |
| Rosa et al., 2007 | 11 |
| S. Grenier, 2008 | 21 |
| Slotema et al., 2011 | 62 |
| Slotema et al., 2012 | 23 |
| Vercammen et al., 2009 | 38 |
| Vercammen et al., 2010 | 18 |
| Total Studies=15 | **Total Randomised=423** |
| Psychosurgery | |
| Zhou et al., 2004 | 36 |
| Total Studies=1 | **Total Randomised=36** |
| Transcranial direct-current stimulation | |
| Fitzgerald, 2007 | 100* |
| Palm, 2011 | 20* |
| Total Studies=2 | **Total Randomised=120** |
| Intravascular irradiation | |
| Wang et al., 2003 | 60 |
| Total Studies=1 | **Total Randomised=60** |
| Haemodialysis | |
| Schulman et al., 1983 | 11 |
| Total Studies=1 | **Total Randomised=11** |

**Pharmacological interventions**

**Table 2 Adjuvant interventions – added to clozapine**

| Study | Number of Patients Randomised |
| --- | --- |
| Antipsychotics | |
| Amisulpride | |
| Assion et al., 2008 | 16 |
| Barnes, 2010 | 230* |
| Genç et al., 2007 | 56 |
| Kreinin et al., 2006 | 20 |
| Total Studies=4 | **Total Randomised=322** |
| Aripiprazole | |
| Barbui et al., 2011 | 106 |
| Chang et al., n.d. | 62 |
| Ding and Li, 2010 | 66 |
| Ma et al., 2007 | 84 |
| Maria Rosaria A Muscatello et al., 2011 | 31 |
| Total Studies=5 | **Total Randomised=349** |
| Fluphenazine | |
| 刘友夺 et al., 2008 | 60 |
| Total Studies=1 | **Total Randomised=60** |
| Haloperidol | |
| Barbui et al., 2011 | 106 |
| Mossaheb et al., 2006 | 10 |
| Total Studies=2 | **Total Randomised=116** |
| Paliperidone | |
| Azcurra, 2011 | 70* |
| Total Studies=1 | **Total Randomised=70** |
| Pipotiazine | |
| Zhu et al., 2002 | 84 |
| Total Studies=1 | **Total Randomised=84** |
| Quetiapine | |
| Genç et al., 2007 | 56 |
| 刘春仙 et al., 2005 | 80 |
| Total Studies=1 | **Total Randomised=136** |
| Risperidone | |
| Honer et al., 2006 | 68 |
| Josiassen et al., 2005 | 40 |
| Kuwilsky et al., 2010 | 24 |
| Peng et al., 2001 | 101 |
| Richardson CM et al., 2009 | 65 |
| Weiner et al., 2010 | 69 |
| 吴丽会, 2002 | 98 |
| 孔庆任 et al., 2001 | 60 |
| 徐儒瑾 et al., 2008 | 37 |
| 陈眷梅 et al., 2003 | 84 |
| Total Studies=10 | **Total Randomised=646** |
| Sertindole | |
| Nielsen et al., 2012 | 50 |
| Total Studies=1 | **Total Randomised=50** |

| Study | Number of Patients Randomised |
| --- | --- |
| Sulpride | |
| 孔庆任 et al., 2001 | 60 |
| 邹果果 et al., 2003 | 90 |
| Total Studies=2 | **Total Randomised=150** |
| Ziprasidone | |
| Kuwilsky et al., 2010 | 24 |
| 任列 et al., 2009 | 60 |
| 莫亚莉, 2010 | 66 |
| Total Studies=3 | **Total Randomised=150** |
| Clozapine added to other antipsychotics | |
| Denney et al., 2001 | 10 |
| 黄祖荣 et al., 2009 | 90 |
| Total Studies=2 | **Total Randomised=100** |
| Antidepressants | |
| Duloxetine | |
| Mico’ et al., 2011 | 33 |
| Total Studies=1 | **Total Randomised=33** |
| Fluvoxamine | |
| Lu et al., 2004 | 68 |
| Total Studies=1 | **Total Randomised=68** |
| Anticonvulsants and mood stabilisers | |
| Valproate | |
| 潘朝霞 et al., 2010 | 34 |
| Total Studies=1 | **Total Randomised=34** |
| Lamotrigine | |
| Tiihonen et al., 2003 | 34 |
| Zoccali et al., 2007 | 51 |
| Total Studies=2 | **Total Randomised=85** |
| Lithium | |
| Small et al., 2003, p. 20 | 20 |
| 孙辉 et al., 2008 | 72 |
| 陈列 et al., 2001 | 60 |
| Total Studies=3 | **Total Randomised=152** |
| Antimicrobials | |
| Inosine | |
| 邓世平 et al., 2006 | 66 |
| Total Studies=1 | **Total Randomised=66** |
| Herbal | |
| Ginkgo Biloba | |
| Doruk et al., 2008 | 42 |
| Total Studies=1 | **Total Randomised=42** |
| Anti-diabetic | |
| Metformin | |
| Carrizo et al., 2009 | 61 |
| Total Studies=1 | **Total Randomised=61** |
| Selective norepinephrine reuptake inhibitor | |
| Atomexetine | |
| Shekhar, 2005 | 126* |
| Total Studies=1 | **Total Randomised=126** |

| Study | Number of Patients Randomised |
| --- | --- |

| Drugs used in Dementia | |
| --- | --- |
| Memantine | |
| de Lucena et al., 2009 | 21 |
| Total Studies=1 | **Total Randomised=21** |
| Amino acids | |
| Glycine | |
| Diaz et al., 2005 | 12 |
| Potkin et al., 1999 | 19 |
| Total Studies=2 | **Total Randomised=31** |

**Table 3 Adjuvant interventions – added to antipsychotics other than clozapine**

| Study | Number of Patients Randomised |
| --- | --- |
| Added to specific antipsychotic | |
| Chlorpromazine added to Olanzapine | |
| 郭建雄 et al., 2003 | 39 |
| Total Studies=1 | **Total Randomised=39** |
| Sulpride added to Olanzapine | |
| 邬德纯 et al., 2005 | 97 |
| Kotler et al., 2004 | 17 |
| Total Studies=2 | **Total Randomised=114** |
| Quetiapine added to Risperidone | |
| 王敬龙 and 王建平, 2010 | 144 |
| Total Studies=1 | **Total Randomised=144** |
| LDOPA added to Chlorpromazine | |
| Owens et al., 1994 | 8 |
| Total Studies=1 | **Total Randomised=8** |
| Ondansetron added to Haloperidol | |
| Zhang et al., 2006 | 121 |
| Total Studies=1 | **Total Randomised=121** |
| Added to unspecified antipsychotics | |
| Amino Acids | |
| Glycine added to unspecified antipsychotics | |
| Heresco-Levy et al., 1996 | 11 |
| Heresco-Levy et al., 1999 | 22 |
| Heresco-Levy et al., 2004 | 17 |
| Total Studies=3 | **Total Randomised=50** |
| Serine added to unspecified antipsychotics | |
| Heresco-Levy et al., 2005 | 39 |
| Total Studies=1 | **Total Randomised=39** |
| Anti-depressants | |
| Citalopram added to unspecified antipsychotics | |
| Ungvari, 1997 | 18 |
| Total Studies=1 | **Total Randomised=18** |
| Escitalopram added to unspecified antipsychotics | |
| Bugarski-Kirola, 2005 | 30* |
| Total Studies=1 | **Total Randomised=30** |
| Mianserin added to unspecified antipsychotics | |
| Shiloh et al., 2002 | 18 |
| Total Studies=1 | **Total Randomised=18** |
| Sertraline added to unspecified antipsychotics | |
| Kim and Woo, 1997 | 77 |
| Total Studies=1 | **Total Randomised=77** |
| Anticonvulsants and mood stabilisers | |
| Valproate added to unspecified antipsychotics | |
| 王刚平 et al., 2009 | 60 |
| 贡永宁 and 郭平, 2010 | 80 |
| Total Studies=2 | **Total Randomised=140** |
| Carbamezapine added to unspecified antipsychotics | |
| Meszaros and Simhandl, 1990 | 24 |
| Neppe, 1983 | 13 |
| Simhandl et al., 1996 | 42 |
| Total Studies=3 | **Total Randomised=79** |
| Study | **Number of Patients Randomised** |
| Topiramate added to unspecified antipsychotics | |
| M R A Muscatello et al., 2011 | 60 |
| Tiihonen, J et al., 2005 | 26 |
| Total Studies=2 | **Total Randomised=86** |
| Lamotrigine added to unspecified antipsychotics | |
| Kremer et al., 2004 | 38 |
| Total Studies=1 | **Total Randomised=38** |
| Lithium added to unspecified antipsychotics | |
| Meszaros et al., 1991 | 19 |
| Schulz et al., 1999 | 41 |
| Simhandl et al., 1996 | 42 |
| Wilson, 1993 | 21 |
| 何燕飞 et al., 2010 | 92 |
| 王强, 1995 | 12 |
| Total Studies=5 | **Total Randomised=227** |
| Antimicrobials | |
| D-cycloserine added to unspecified antipsychotics | |
| Heresco-Levy et al., 1998 | 9 |
| Heresco-Levy et al., 2005 | 24 |
| Total Studies=2 | **Total Randomised=35** |
| Ketoconazole added to unspecified antipsychotics | |
| Marco et al., 2002, p. 199 | 15 |
| Owen, 1996 | 9 |
| Total Studies=2 | **Total Randomised=24** |
| Herbal | |
| Ning Xin Tang added to unspecified antipsychotics | |
| 段武钢 et al., 2010 | 60 |
| Total Studies=1 | **Total Randomised=60** |
| Ginkgo Biloba added to unspecified antipsychotics | |
| Zhang et al., 2001b | 82 |
| Total Studies=1 | **Total Randomised=82** |
| Yi Gan San added to unspecified antipsychotics | |
| Miyaoka et al., 2009 | 59 |
| Total Studies=1 | **Total Randomised=59** |
| Movement Disorder Drugs | |
| Apomorphine added to unspecified antipsychotics | |
| Tamminga et al., 1978 | 18 |
| Total Studies=1 | **Total Randomised=18** |
| Tetrabenazine added to unspecified antipsychotics | |
| Remington et al., 2012 | 41 |
| Total Studies=1 | **Total Randomised=41** |
| Opioids | |
| Methadone added to unspecified antipsychotics | |
| Brizer et al., 1985 | 7 |
| Total Studies=1 | **Total Randomised=7** |
| Salt or ester of benzoic acid | |
| Benzoate added to unspecified antipsychotics | |
| Lane, 2011 | 60* |
| Total Studies=1 | **Total Randomised=60** |
| Histamine H2 receptor antagonist | |
| Famotidine added to unspecified antipsychotics | |
| Meskanen et al., 2013 | 30 |
| Total Studies=1 | **Total Randomised=30** |
| Study | **Number of Patients Randomised** |
| Oestrogen receptor antagonist | |
| Tamoxifem added to unspecified antipsychotics | |
| Lieberman, 2009 | 26 |
| Total Studies=2 | **Total Randomised=26** |

**Table 4 Non-adjuvant use of antipsychotic medication**

| Study | Number of Patients Randomised |
| --- | --- |
| Amisulpride | |
| Amisulpride vs antipsychotics which include clozapine | |
| Krivoy, 2011 | 140* |
| Total Studies=1 | **Total Randomised=140** |
| Aripiprazole | |
| Aripiprazole vs antipsychotics which include clozapine | |
| 于彩霞 et al., 2006 | 48 |
| 张仁凯 et al., 2008 | 78 |
| 张启林 and 翟江, 2010 | 120 |
| 张星亮 et al., 2012 | 68 |
| 张艳琦 and 于振东, 2009 | 72 |
| 李爱凤 et al., 2007 | 65 |
| 蒋国庆 and 罗捷, 2009 | 40 |
| 闫宝昌, 2008 | 100 |
| Total Studies=8 | **Total Randomised=591** |
| Aripiprazole vs antipsychotics excluding clozapine | |
| Kane et al., 2007 | 300 |
| 刘伟锋 and 张卫敏, 2008 | 63 |
| 朱琛擘 et al., 2008 | 78 |
| 毛星 et al., 2010 | 70 |
| Total Studies=4 | **Total Randomised=511** |
| Clozapine | |
| Dosing levels | |
| Abraham et al., 1997 | 30 |
| Nair et al., 1997 | 42 |
| Potkin et al., 1994 | 58 |
| Simpson et al., 1999 | 50 |
| VanderZwaag et al., 1996 | 56 |
| Total Studies=5 | **Total Randomised=236** |
| Clozapine vs non-clozapine antipsychotics | |
| Andreoli et al., 1996 | 30 |
| Bitter et al., 2004 | 150 |
| Bondolfi et al., 1996 | 86 |
| Bustillo et al., 1996 | 39 |
| Chengappa, 2001 | 180* |
| Chouinard et al., 1994 | 11 |
| Chowdhury et al., 1999 | 60 |
| Claghorn et al., 1987 | 151 |
| Conley et al., 1999 | 27 |
| Conley et al., 2003 | 13 |
| Daniel, 1994 | 20 |
| Estrella et al., 1996 | 22 |
| Flynn et al., 1998 | 86 |
| Green, 2005 | 35* |
| Green, 2005 | 66* |
| Hong et al., 1997 | 40 |
| Jayathilake et al., 2005 | 46 |
| Study | **Number of Patients Randomised** |
| Kane et al., 1988 | 268 |
| Kane et al., 2001 | 71 |
| Kenny and Meltzer, 1992 | 37 |
| Konrad et al., 1997 | 61 |
| Krivoy, 2011 | 140* |
| Kumra et al., 1996 | 21 |
| Kumra et al., 2008 | 39 |
| Lee et al., 1994 | 83 |
| Lee et al., 1995 | 33 |
| Lewis et al., 2006 | 136 |
| Lieberman, 2001 | 224* |
| Lindenmayer et al., 1996 | 28 |
| Louwerens et al., 2000 | 36 |
| McEvoy et al., 2006 | 99 |
| McGurk et al., 2005 | 97 |
| Meltzer et al., 2008 | 40 |
| Pickar et al., 1992 | 21 |
| Potkin et al., 2003 | 15 |
| Rosenheck et al., 1995 | 423 |
| Sacchetti et al., 2009 | 147 |
| Schulz et al., 1999 | 40 |
| Shaw et al., 2006 | 25 |
| Tollefson et al., 2001 | 180 |
| Volavka et al., 2002 | 157 |
| Wahlbeck et al., 2000 | 21 |
| 于彩霞 et al., 2006 | 48 |
| 任进军 and 苏俊玲, 2000 | 48 |
| 余国汉 and 黄雄, 1999 | 135 |
| 冯雪雁 and 彭华, 2002 | Unavailable |
| 刘佰聪, 2007 | 98 |
| 刘英, 2005 | 35 |
| 唐振坤 et al., 2007 | 76 |
| 唐步春 et al., 2005 | 133 |
| 孙祺章 et al., 2001 | 64 |
| 张仁凯 et al., 2008 | 78 |
| 张启林 and 翟江, 2010 | 120 |
| 张建新 et al., 2005 | 70 |
| 张星亮 et al., 2012 | 68 |
| 张艳琦 and 于振东, 2009 | 72 |
| 张艳琦 and 于振东, 2010 | 80 |
| 曹红军 et al., 2003 | 60 |
| 朱倩芸 et al., 2003 | 30 |
| 朱凤艳 et al., 2003 | 72 |
| 李晓菊 et al., 2009 | 80 |
| 李爱凤 et al., 2007 | 65 |
| 杨彦林, 2006 | 68 |
| 杨甫德 et al., 2005 | 60 |
| Study | **Number of Patients Randomised** |
| 杨福收 et al., 2004 | 56 |
| 杨立身 et al., 2005 | 43 |
| 林卓毅 et al., 2009 | 85 |
| 王淑芳 and 邢君, 2010 | 144 |
| 王秀梅 et al., 2010 | 98 |
| 王艳君, 2007 | 70 |
| 王荣芝 et al., 2002 | 70 |
| 程绍忠 et al., 2002 | 40 |
| 蒋国庆 and 罗捷, 2009 | 40 |
| 郑晖 and 许崇涛, 2001 | 36 |
| 闫宝昌, 2008 | 100 |
| 阮江红 and 黄国红, 2005 | 60 |
| 陈丽霞 and 田常青, 2010 | 60 |
| 韩鹏, 2000 | 70 |
| 马迅, 2010 | 70 |
| 高军 and 张伟, 2010 | 65 |
| 高军 and 张文兵, 2006 | 64 |
| 高桂林 and 李翠茹, 2006 | 64 |
| Total Studies=82 | **Total Randomised=6299** |
| Olanzapine | |
| Olanzapine vs antipsychotics which include clozapine | |
| Bitter et al., 2004 | 150 |
| Conley et al., 1999 | 27 |
| Conley et al., 2003 | 13 |
| Green, 2005 | 35* |
| Jayathilake et al., 2005 | 46 |
| Kumra et al., 2008 | 39 |
| Lewis et al., 2006 | 136 |
| McEvoy et al., 2006 | 99 |
| Meltzer et al., 2008 | 40 |
| Shaw et al., 2006 | 25 |
| Tollefson et al., 2001 | 180 |
| Volavka et al., 2002 | 157 |
| 唐振坤 et al., 2007 | 76 |
| 李晓菊 et al., 2009 | 80 |
| 杨立身 et al., 2005 | 43 |
| 王艳君, 2007 | 70 |
| 郑晖 and 许崇涛, 2001 | 36 |
| 高军 and 张伟, 2010 | 65 |
| 高桂林 and 李翠茹, 2006 | 64 |
| Total Studies=19 | **Total Randomised=1381** |
| Olanzapine vs antipsychotics which exclude clozapine | |
| Altamura et al., 2002 | 24 |
| Breier and Hamilton, 1999 | 526 |
| Buchanan et al., 2005 | 63 |
| Conley et al., 1998 | 84 |
| Study | **Number of Patients Randomised** |
| Smith et al., 2001 | 34 |
| Zhu and Pi, 2012 | 30 |
| 毛星 et al., 2010 | 70 |
| 王立钢 et al., 2004 | 68 |
| Total Studies=8 | **Total Randomised=899** |
| Risperidone | |
| Risperidone vs antipsychotics which include clozapine | |
| Bondolfi et al., 1996 | 86 |
| Chengappa, 2001 | 180* |
| Chouinard et al., 1994 | 11 |
| Chowdhury et al., 1999 | 60 |
| Daniel, 1994 | 20 |
| Estrella et al., 1996 | 22 |
| Flynn et al., 1998 | 86 |
| Konrad et al., 1997 | 61 |
| Lewis et al., 2006 | 136 |
| Lieberman, 2001 | 224* |
| Lindenmayer et al., 1996 | 28 |
| McEvoy et al., 2006 | 99 |
| McGurk et al., 2005 | 97 |
| Volavka et al., 2002 | 157 |
| Wahlbeck et al., 2000 | 21 |
| 任进军 and 苏俊玲, 2000 | 48 |
| 余国汉 and 黄雄, 1999 | 135 |
| 冯雪雁 and 彭华, 2002 | Unavailable |
| 刘英, 2005 | 35 |
| 孙祺章 et al., 2001 | 64 |
| 张建新 et al., 2005 | 70 |
| 曹红军 et al., 2003 | 60 |
| 朱倩芸 et al., 2003 | 30 |
| 朱凤艳 et al., 2003 | 72 |
| 杨甫德 et al., 2005 | 60 |
| 王淑芳 and 邢君, 2010 | 144 |
| 王荣芝 et al., 2002 | 70 |
| 程绍忠 et al., 2002 | 40 |
| 陈丽霞 and 田常青, 2010 | 60 |
| 韩鹏, 2000 | 70 |
| 马迅, 2010 | 70 |
| 高军 and 张文兵, 2006 | 64 |
| Total Studies=32 | **Total Randomised=2380** |
| Risperidone vs antipsychotics which exclude clozapine | |
| Ames et al., 1997 | 67 |
| Beck et al., 1997 | 20 |
| Conley et al., 2005 | 52 |
| Kane et al., 2011 | 321 |
| Liberman et al., 2002 | 36 |
| Mercer et al., 1997 | 43 |
| Zhang et al., 2001a | 78 |
| Study | **Number of Patients Randomised** |
| 冯春霞 et al., 2001 | 70 |
| 刘伟锋 and 张卫敏, 2008 | 63 |
| 刘绍梅 et al., 2001 | Unavailable |
| 吴树靖 and 邢桂芳, 2002 | 70 |
| 朱华芳, 2007 | 84 |
| 王勇 et al., 2010 | 120 |
| 郑占杰 et al., 2003 | 84 |
| Total Studies=14 | **Total Randomised=1108** |
| Risperidone dosing studies | |
| 杨立身 et al., 2004 | 27 |
| Total Studies=1 | **Total Randomised=27** |
| Quetiapine | |
| Quetiapine vs antipsychotics which include clozapine | |
| Lewis et al., 2006 | 136 |
| McEvoy et al., 2006 | 99 |
| 刘佰聪, 2007 | 98 |
| 杨彦林, 2006 | 68 |
| 杨福收 et al., 2004 | 56 |
| 王秀梅 et al., 2010 | 98 |
| 阮江红 and 黄国红, 2005 | 60 |
| Total Studies=7 | **Total Randomised=615** |
| Quetiapine vs antipsychotics which exclude clozapine | |
| AstraZeneca, 2000 | 236 |
| Buckley et al., 2004 | 95 |
| Conley et al., 2005 | 52 |
| 王勇 et al., 2010 | 120 |
| Total Studies=4 | **Total Randomised=503** |
| Quetiapine dosing studies | |
| Lindenmayer et al., 2011 | 60 |
| Total Studies=1 | **Total Randomised=60** |
| Sertindole | |
| Sertindole vs antipsychotics which exclude clozapine | |
| Kane et al., 2011 | 321 |
| Total Studies=1 | **Total Randomised=321** |
| Ziprasidone | |
| Ziprasidone vs antipsychotics which include clozapine | |
| Sacchetti et al., 2009 | 147 |
| 唐步春 et al., 2005 | 133 |
| 张艳琦 and 于振东, 2010 | 80 |
| 林卓毅 et al., 2009 | 85 |
| Total Studies=4 | **Total Randomised=445** |
| Ziprasidone vs antipsychotics which exclude clozapine | |
| Kane et al., 2006 | 306 |
| Total Studies=1 | **Total Randomised=306** |
| Chlorpromazine | |
| Chlorpromazine vs antipsychotics which include clozapine | |
| Claghorn et al., 1987 | 151 |
| Green, 2005 | 66* |
| Hong et al., 1997 | 40 |
| Study | **Number of Patients Randomised** |
| Kane et al., 1988 | 268 |
| Total Studies=4 | **Total Randomised=525** |
| Chlorpromazine vs antipsychotics which exclude clozapine | |
| AstraZeneca, 2000 | 236 |
| Conley et al., 1998 | 84 |
| Kane et al., 2006 | 306 |
| Lal et al., 2006 | 38 |
| McCreadie and MacDonald, 1977 | 20 |
| Mercer et al., 1997 | 43 |
| 冯春霞 et al., 2001 | 70 |
| 吴树靖 and 邢桂芳, 2002 | 70 |
| 朱华芳, 2007 | 84 |
| 王立钢 et al., 2004 | 68 |
| 郑占杰 et al., 2003 | 84 |
| Total Studies=10 | **Total Randomised=1019** |
| Fluphenazine | |
| Dosing levels | |
| Quitkin et al., 1975 | 31 |
| Total Studies=1 | **Total Randomised=31** |
| Fluphenazine vs antipsychotics which include clozapine | |
| Pickar et al., 1992 | 21 |
| Total Studies=1 | **Total Randomised=21** |
| Fluphenazine vs antipsychotics which exclude clozapine | |
| Conley et al., 2005 | 52 |
| Hall et al., 1968 | 50 |
| Kane et al., 1993 | 83 |
| 刘绍梅 et al., 2001 | Unavailable |
| Total Studies=4 | **Total Randomised=185** |
| Haloperidol | |
| Haloperidol vs antipsychotics which include clozapine | |
| Bustillo et al., 1996 | 39 |
| Kane et al., 2001 | 71 |
| Kumra et al., 1996 | 21 |
| Lee et al., 1995 | 33 |
| Lieberman, 2001 | 224* |
| Potkin et al., 2003 | 15 |
| Rosenheck et al., 1995 | 423 |
| Volavka et al., 2002 | 157 |
| Total Studies=8 | **Total Randomised=983** |
| Haloperidol vs antipsychotics which exclude clozapine | |
| Altamura et al., 2002 | 24 |
| Ames et al., 1997 | 67 |
| Breier and Hamilton, 1999 | 526 |
| Buchanan et al., 2005 | 63 |
| Buckley et al., 2004 | 95 |
| Conley et al., 1993 | 16 |
| Gonier et al., 1970 | 40 |
| Hall et al., 1968 | 50 |
| Kane et al., 1993 | 83 |
| Liberman et al., 2002 | 36 |
| McCreadie and MacDonald, 1977 | 20 |
| Study | **Number of Patients Randomised** |
| Smith et al., 2001 | 34 |
| Zhang et al., 2001a | 78 |
| 朱华芳, 2007 | 84 |
| 朱琛擘 et al., 2008 | 78 |
| Total Studies=15 | **Total Randomised=1294** |
| Haloperidol dosing studies | |
| Browne et al., 1988 | 11 |
| Total Studies=1 | **Total Randomised=11** |
| Perphenazine | |
| Perphenazine vs antipsychotics which exclude clozapine | |
| Kane et al., 2007 | 300 |
| Total Studies=1 | **Total Randomised=300** |
| Thiothexene | |
| Thiothexene dosing studies | |
| Gardos et al., 1974 | 40 |
| Hollister et al., 1987 | 42 |
| Total Studies=2 | **Total Randomised=82** |
| Others** | |
| Others dosing studies | |
| McCreadie et al., 1979 | 23 |
| Meltzer, 2012 | 120* |
| Total Studies=2 | **Total Randomised=143** |
| Others vs antipsychotics which exclude clozapine | |
| Gardos et al., 1978 | 19 |
| Lal et al., 2006 | 38 |
| Vital-Herne et al., 1986 | 7 |
| Zhu and Pi, 2012 | 30 |
| Total Studies=4 | **Total Randomised=94** |

*Denotes unreported trials

**Flupenthixol, Levopromazine. Lurasidone, Mesoridazine, Paliperidone, Remoxipride, Thioridazine

**References**

Abraham, G., Nair, C., Tracy, J.I., Simpson, G.M., Josiassen, R.C., 1997. The effects of clozapine on symptom clusters in treatment-refractory patients. J. Clin. Psychopharmacol. 17, 49–53.

Altamura, A.., Velonà, I., Curreli, R., Mundo, E., Bravi, D., 2002. Is olanzapine better than haloperidol in resistant schizophrenia? A double-blind study in partial responders. Int. J. Psychiatry Clin. Pract. 6, 107–111. doi:10.1080/136515002753724117

Ames, D., Wirsching, W., Marshall, B., Green, M., McGurk, S., Mintz, J., Marder, S.R., 1997. Treatment-resistant schizophrenia: efficacy of risperidone versus haloperidol, in: 150th Annual Meeting of the American Psychiatric Association. San Diego, California, USA.

Andreoli, A., Kolatte, E., Eisele, R., Schneider, E., Liechti, A., 1996. Clozapine efficacy and cost-effectiveness in treatment resistant schizophrenic outpatients, in: 10th World Congress of Psychiatry. Madrid Spain.

Assion, H.-J., Reinbold, H., Lemanski, S., Basilowski, M., Juckel, G., 2008. Amisulpride augmentation in patients with schizophrenia partially responsive or unresponsive to clozapine. A randomized, double-blind, placebo-controlled trial. Pharmacopsychiatry 41, 24–28. doi:10.1055/s-2007-993209

AstraZeneca, 2000. A multicentre, double-blind, randomised trial to compare the effects of seroquel and chlorpromazine in patients with treatment resistant schizophrenia (5077IL/0054 TRESS ).

Azcurra, D., 2011. NCT01279213 Clozapine/Paliperidone Versus Clozapine/Placebo in Resistant Schizophrenia - Full Text View - ClinicalTrials.gov [WWW Document]. URL http://clinicaltrials.gov/show/ (accessed 3.18.14).

Barbui, C., Accordini, S., Nosè, M., Stroup, S., Purgato, M., Girlanda, F., Esposito, E., Veronese, A., Tansella, M., Cipriani, A., CHAT (Clozapine Haloperidol Aripiprazole Trial) Study Group, 2011. Aripiprazole versus haloperidol in combination with clozapine for treatment-resistant schizophrenia in routine clinical care: a randomized, controlled trial. J. Clin. Psychopharmacol. 31, 266–273. doi:10.1097/JCP.0b013e318219cba3

Barnes, T., 2010. ISRCTN68824876 - Amisulpride augmentation in clozapine-unresponsive schizophrenia [WWW Document]. URL http://www.controlled-trials.com/ISRCTN68824876 (accessed 3.18.14).

Beck, N.C., Greenfield, S.R., Gotham, H., Menditto, A.A., Stuve, P., Hemme, C.A., 1997. Risperidone in the management of violent, treatment-resistant schizophrenics hospitalized in a maximum security forensic facility. J. Am. Acad. Psychiatry Law 25, 461–468.

Birchwood, M., Peters, E., Tarrier, N., Dunn, G., Lewis, S., Wykes, T., Davies, L., Lester, H., Michail, M., 2011. A multi-centre, randomised controlled trial of cognitive therapy to prevent harmful compliance with command hallucinations. BMC Psychiatry 11, 155. doi:10.1186/1471-244X-11-155

Bitter, I., Dossenbach, M.R.K., Brook, S., Feldman, P.D., Metcalfe, S., Gagiano, C.A., Füredi, J., Bartko, G., Janka, Z., Banki, C.M., Kovacs, G., Breier, A., Olanzapine HGCK Study Group, 2004. Olanzapine versus clozapine in treatment-resistant or treatment-intolerant schizophrenia. Prog. Neuropsychopharmacol. Biol. Psychiatry 28, 173–180. doi:10.1016/j.pnpbp.2003.09.033

Blumberger, D.M., Christensen, B.K., Zipursky, R.B., Moller, B., Chen, R., Fitzgerald, P.B., Daskalakis, Z.J., 2012. MRI-targeted repetitive transcranial magnetic stimulation of Heschl’s gyrus for refractory auditory hallucinations. Brain Stimulat. 5, 577–585. doi:10.1016/j.brs.2011.12.002

Bondolfi, G., Baumann, P., Dufour, H., 1996. Treatment-resistant schizophrenia: clinical experience with new antipsychotics. Eur. Neuropsychopharmacol. J. Eur. Coll. Neuropsychopharmacol. 6 Suppl 2, S21–25.

Braga, R., 2009. A Randomized Controlled Trial of ECT In Clozapine-Refractory Schizophrenia. Biol. Psychiatry 65, S212–3.

Breier, A., Hamilton, S.H., 1999. Comparative efficacy of olanzapine and haloperidol for patients with treatment-resistant schizophrenia. Biol. Psychiatry 45, 403–411.

Brizer, D.A., Hartman, N., Sweeney, J., Millman, R.B., 1985. Effect of methadone plus neuroleptics on treatment-resistant chronic paranoid schizophrenia. Am. J. Psychiatry 142, 1106–1107.

Browne, F.W.A., Cooper, S.J., Wilson, R., King, D.J., 1988. Serum haloperidol levels and clinical response in chronic, treatment-resistant schizophrenic patients. J. Psychopharmacol. (Oxf.) 2, 94–103. doi:10.1177/026988118800200204

Brunelin, J., Poulet, E., Bediou, B., Kallel, L., Dalery, J., D’amato, T., Saoud, M., 2006. Low frequency repetitive transcranial magnetic stimulation improves source monitoring deficit in hallucinating patients with schizophrenia. Schizophr. Res. 81, 41–45. doi:10.1016/j.schres.2005.10.009

Buchain, P.C., Vizzotto, A.D.B., Henna Neto, J., Elkis, H., 2003. Randomized controlled trial of occupational therapy in patients with treatment-resistant schizophrenia. Rev. Bras. Psiquiatr. São Paulo Braz. 1999 25, 26–30.

Buchanan, R.W., Ball, M.P., Weiner, E., Kirkpatrick, B., Gold, J.M., McMahon, R.P., Carpenter, W.T., Jr, 2005. Olanzapine treatment of residual positive and negative symptoms. Am. J. Psychiatry 162, 124–129. doi:10.1176/appi.ajp.162.1.124

Buckley, P.F., Goldstein, J.M., Emsley, R.A., 2004. Efficacy and tolerability of quetiapine in poorly responsive, chronic schizophrenia. Schizophr. Res. 66, 143–150. doi:10.1016/j.schres.2003.06.001

Bugarski-Kirola, D., 2005. NCT00231335: Efficacy and Safety Study of Escitalopram Augmentation in Treatment Resistant Schizophrenia [WWW Document]. URL http://clinicaltrials.gov/ct2/show/NCT00231335 (accessed 3.25.14).

Bustillo, J.R., Buchanan, R.W., Irish, D., Breier, A., 1996. Differential effect of clozapine on weight: a controlled study. Am. J. Psychiatry 153, 817–819.

Cai, X., 2008. A comparison study of MECT combining clozapine in the treatment of treatment - resistant schizophrenia. Med. J. Chin. Peoples Health 20, 1423–4.

Carrizo, E., Fernández, V., Connell, L., Sandia, I., Prieto, D., Mogollón, J., Valbuena, D., Fernández, I., de Baptista, E.A., Baptista, T., 2009. Extended release metformin for metabolic control assistance during prolonged clozapine administration: a 14 week, double-blind, parallel group, placebo-controlled study. Schizophr. Res. 113, 19–26. doi:10.1016/j.schres.2009.05.007

Chang, J.S., Ahn, Y.M., Park, H.J., Lee, K.Y., Kim, S.H., Kang, U.G., Kim, Y.S., n.d. Aripiprazole Augmentation in Clozapine-Treated Patients With Refractory Schizophrenia : An 8-Week, Randomized, Double-Blind, Placebo-Controlled Trial. J. Clin. Psychiatry 69, 720–731.

Chanpattana, W., Chakrabhand, M.L., Sackeim, H.A., Kitaroonchai, W., Kongsakon, R., Techakasem, P., Buppanharun, W., Tuntirungsee, Y., Kirdcharoen, N., 1999. Continuation ECT in treatment-resistant schizophrenia: a controlled study. J. ECT 15, 178–192.

Chengappa, K., 2001. New antipsychotics-clinical trials and followup.

Chouinard, G., Vainer, J.L., Bélanger, M.C., Turnier, L., Beaudry, P., Roy, J.Y., Miller, R., 1994. Risperidone and clozapine in the treatment of drug-resistant schizophrenia and neuroleptic-induced supersensitivity psychosis. Prog. Neuropsychopharmacol. Biol. Psychiatry 18, 1129–1141.

Chowdhury, A., Mukherjee, A., Ghosh, K., Chowdhury, S., Das Sen, K., 1999. Horizon of a new hope: recovery of schizophrenia in India. Int. Med. J. 6, 181–5.

Claghorn, J., Honigfeld, G., Abuzzahab, F.S., Sr, Wang, R., Steinbook, R., Tuason, V., Klerman, G., 1987. The risks and benefits of clozapine versus chlorpromazine. J. Clin. Psychopharmacol. 7, 377–384.

Clarke, S., 2011. A randomised control trial comparing the effectiveness of acceptance and commitment therapy with treatment as usual, cognitive behavioural therapy, with treatment resistant participants.

Conley, R., Tamminga, C., An Nguyen, J., Hain, R., 1993. Remoxipride therapy in treatment resistant schizophrenia. Schizophr. Res. 9, 235–236.

Conley, R.R., Kelly, D.L., Nelson, M.W., Richardson, C.M., Feldman, S., Benham, R., Steiner, P., Yu, Y., Khan, I., McMullen, R., Gale, E., Mackowick, M., Love, R.C., 2005. Risperidone, quetiapine, and fluphenazine in the treatment of patients with therapy-refractory schizophrenia. Clin. Neuropharmacol. 28, 163–168.

Conley, R.R., Kelly, D.L., Richardson, C.M., Tamminga, C.A., Carpenter, W.T.J., 2003. The Efficacy of High-Dose Olanzapine Versus Clozapine in Treatment-Resistant Schizophrenia: A Double-Blind Crossover Study. J. Clin. Psychopharmacol. 23.

Conley, R.R., Tamminga, C.A., Bartko, J.J., Richardson, C., Peszke, M., Lingle, J., Hegerty, J., Love, R., Gounaris, C., Zaremba, S., 1998. Olanzapine compared with chlorpromazine in treatment-resistant schizophrenia. Am. J. Psychiatry 155, 914–920.

Conley, R.R., Tamminga, C.A., Kelly, D.L., Richardson, C.M., 1999. Treatment-resistant schizophrenic patients respond to clozapine after olanzapine non-response. Biol. Psychiatry 46, 73–77.

Daniel, D., 1994. Crossover comparison of risperidone and clozapine on clinical, cognitive, and side effect measures in treatment-resistant psychosis. Psychopharmacol. Bull. 30, 629.

Daskalakis, Z., 2007. Repetitive transcranial magnetic stimulation (RTMS) in the neurophysiology and treatment of schizophrenia. Schizophr. Bull. 33, 402.

De Lucena, D., Fernandes, B.S., Berk, M., Dodd, S., Medeiros, D.W., Pedrini, M., Kunz, M., Gomes, F.A., Giglio, L.F., Lobato, M.I., Belmonte-de-Abreu, P.S., Gama, C.S., 2009. Improvement of negative and positive symptoms in treatment-refractory schizophrenia: a double-blind, randomized, placebo-controlled trial with memantine as add-on therapy to clozapine. J. Clin. Psychiatry 70, 1416–1423. doi:10.4088/JCP.08m04935gry

De Paiva Barretto, E.M., Kayo, M., Avrichir, B.S., Sa, A.R., Camargo, M. das G.M., Napolitano, I.C., Nery, F.G., Pinto, J.A., Bannwart, S., Scemes, S., Di Sarno, E., Elkis, H., 2009. A Preliminary Controlled Trial of Cognitive Behavioral Therapy in Clozapine-Resistant Schizophrenia: J. Nerv. Ment. Dis. 197, 865–868. doi:10.1097/NMD.0b013e3181be7422

Denney, D., Stevens, J., Wilson, W., 2001. Low dose clozapine treatment of drug resistant schizophrenia. Schizophr. Res. 49, 225.

Diaz, P., Bhaskara, S., Dursun, S.M., Deakin, B., 2005. Double-blind, placebo-controlled, crossover trial of clozapine plus glycine in refractory schizophrenia negative results. J. Clin. Psychopharmacol. 25, 277–278.

Ding, Y.F., 2007. Risperidone and electric convulsive term efficacy of the treatment of refractory schizophrenia control study. Shandong Arch. Psychiatry 20, 397–8.

Ding, Z., Li, T., 2010. A study of Aripiprazole combined with Clozapine in refractory schizophrenia. Med. J. Chin. People’s Health 22, 1780–1781.

Doruk, A., Uzun, O., Ozşahin, A., 2008. A placebo-controlled study of extract of ginkgo biloba added to clozapine in patients with treatment-resistant schizophrenia. Int. Clin. Psychopharmacol. 23, 223–227. doi:10.1097/YIC.0b013e3282fcff2f

Durham, R.C., Guthrie, M., Morton, R.V., Reid, D.A., Treliving, L.R., Fowler, D., Macdonald, R.R., 2003. Tayside—Fife clinical trial of cognitive—behavioural therapy for medication-resistant psychotic symptoms Results to 3-month follow-up. Br. J. Psychiatry 182, 303–311. doi:10.1192/bjp.2.360

Estrella, M., Soria, F., Gonzalez, C., 1996. Cost-effectiveness of clozapine vs respiridone for treatment-resistant schizophrenic patients, in: 10th World Congress of Psychiatry. Madrid Spain.

Fitzgerald, P.B., 2007. A Double Blind Sham Controlled Trial of tDCS in Treating Schizophrenia and Depression - Clinical Trials Studies [WWW Document]. URL http://clinical-trials.findthebest.com/l/30087/A-Double-Blind-Sham-Controlled-Trial-of-tDCS-in-Treating-Schizophrenia-and-Depression (accessed 3.18.14).

Fitzgerald, P.B., Benitez, J., Daskalakis, J.Z., Brown, T.L., Marston, N.A.U., de Castella, A., Kulkarni, J., 2005. A double-blind sham-controlled trial of repetitive transcranial magnetic stimulation in the treatment of refractory auditory hallucinations. J. Clin. Psychopharmacol. 25, 358–362.

Fitzgerald, P.B., Herring, S., Hoy, K., McQueen, S., Segrave, R., Kulkarni, J., Daskalakis, Z.J., 2008. A study of the effectiveness of bilateral transcranial magnetic stimulation in the treatment of the negative symptoms of schizophrenia. Brain Stimulat. 1, 27–32. doi:10.1016/j.brs.2007.08.001

Flynn, S.W., MacEwan, G.W., Altman, S., Kopala, L.C., Fredrikson, D.H., Smith, G.N., Honer, W.G., 1998. An open comparison of clozapine and risperidone in treatment-resistant schizophrenia. Pharmacopsychiatry 31, 25–29. doi:10.1055/s-2007-979291

Freeman, D., Garety, P., Fowler, D., Kuipers, E., Dunn, G., Bebbington, P., Hadley, C., 1998. The London-East Anglia randomized controlled trial of cognitive-behaviour therapy for psychosis. IV: Self-esteem and persecutory delusions. Br. J. Clin. Psychol. Br. Psychol. Soc. 37 ( Pt 4), 415–430.

Gao, J., Zhang, W., 2009. Modif ied electroconvulsive treatment of refractory schizophrenia. 现代药物与临床 27, 577–8.

Gardos, G., Orzack, M.H., Finn, G., Cole, J.O., 1974. High and low dose thiothixene treatment in chronic schizophrenia. Dis. Nerv. Syst. 35, 53–58.

Gardos, G., Tecce, J.J., Hartmann, E., Bowers, P., Cole, J.O., 1978. Treatment with mesoridazine and thioridazine in chronic schizophrenia: I. Assessment of clinical and electrophysiologic responses in refractory hallucinating schizophrenics. Compr. Psychiatry 19, 517–525.

Genç, Y., Taner, E., Candansayar, S., 2007. Comparison of clozapine-amisulpride and clozapine-quetiapine combinations for patients with schizophrenia who are partially responsive to clozapine: a single-blind randomized study. Adv. Ther. 24, 1–13.

Goff, D., Cather, C., Gottlieb, J., Macklin, E., 2011. The Potential Role for D-cycloserine Facilitation of CBT in Schizophrenia: Int. Clin. Psychopharmacol. 26, e135. doi:10.1097/01.yic.0000405863.45893.11

Gonier, T., Schiele, B.C., Vestre, N.D., 1970. A comparison of haloperidol and thioridazine HC1 in chronic treatment-resistant schizophrenics. Behav. Neuropsychiatry 2, 47–49 passim.

Goswami, U., Kumar, U., Singh, B., 2003. Efficacy of Electroconvulsive Therapy in Treatment Resistant Schizophreinia : A double-blind study. Indian J. Psychiatry 45, 26–29.

Green, A., 2005. NCT00169065: Effectiveness of Clozapine Versus Olanzapine for Treatment-Resistant Schizophrenia - Full Text View - ClinicalTrials.gov [WWW Document]. URL http://clinicaltrials.gov/ct2/show/NCT00169065 (accessed 3.26.14).

Haddock, G., Slade, P.D., Bentall, R.P., Reid, D., Faragher, E.B., 1998. A comparison of the long-term effectiveness of distraction and focusing in the treatment of auditory hallucinations. Br. J. Med. Psychol. 71 ( Pt 3), 339–349.

Hall, W.B., Vestre, N.D., Schiele, B.C., Zimmermann, R., 1968. A controlled comparison of haloperidol and fluphenazine in chronic treatment-resistant schizophrenics. Dis. Nerv. Syst. 29, 405–408.

Hayward, P., David, A., Green, N., Rabe-Hesketh, S., Haworth, E., Thompson, N., Shaffer, S., Hastilow, S., Wykes, T., 2009. Promoting Therapeutic Alliance in Clozapine Users: An Exploratory Randomized Controlled Trial. Clin. Schizophr. Relat. Psychoses 3, 127–132. doi:10.3371/CSRP.3.3.1

Heresco-Levy, U., Ermilov, M., Lichtenberg, P., Bar, G., Javitt, D.C., 2004. High-dose glycine added to olanzapine and risperidone for the treatment of schizophrenia. Biol. Psychiatry 55, 165–171.

Heresco-Levy, U., Javitt, D.C., Ebstein, R., Vass, A., Lichtenberg, P., Bar, G., Catinari, S., Ermilov, M., 2005. D-serine efficacy as add-on pharmacotherapy to risperidone and olanzapine for treatment-refractory schizophrenia. Biol. Psychiatry 57, 577–585. doi:10.1016/j.biopsych.2004.12.037

Heresco-Levy, U., Javitt, D.C., Ermilov, M., Mordel, C., Horowitz, A., Kelly, D., 1996. Double-blind, placebo-controlled, crossover trial of glycine adjuvant therapy for treatment-resistant schizophrenia. Br. J. Psychiatry J. Ment. Sci. 169, 610–617.

Heresco-Levy, U., Javitt, D.C., Ermilov, M., Mordel, C., Silipo, G., Lichtenstein, M., 1999. Efficacy of high-dose glycine in the treatment of enduring negative symptoms of schizophrenia. Arch. Gen. Psychiatry 56, 29–36.

Heresco-Levy, U., Javitt, D.C., Ermilov, M., Silipo, G., Shimoni, J., 1998. Double-blind, placebo-controlled, crossover trial of D-cycloserine adjuvant therapy for treatment-resistant schizophrenia. Int. J. Neuropsychopharmacol. Off. Sci. J. Coll. Int. Neuropsychopharmacol. CINP 1, 131–135. doi:10.1017/S1461145798001242

Hodel, B., Kern, R.S., Brenner, H.D., 2004. Emotion Management Training (EMT) in persons with treatment-resistant schizophrenia: first results. Schizophr. Res. 68, 107–108. doi:10.1016/S0920-9964(03)00119-1

Hoffman RE, Hawkins KA, Gueorguieva R, et al, 2003. TRanscranial magnetic stimulation of left temporoparietal cortex and medication-resistant auditory hallucinations. Arch. Gen. Psychiatry 60, 49–56. doi:10.1001/archpsyc.60.1.49

Hollister, L.E., Lombrozo, L., Huang, C.C., 1987. Plasma concentrations of thiothixene and clinical response in treatment-resistant schizophrenics. Int. Clin. Psychopharmacol. 2, 77–82.

Honer, W.G., Thornton, A.E., Chen, E.Y.H., Chan, R.C.K., Wong, J.O.Y., Bergmann, A., Falkai, P., Pomarol-Clotet, E., McKenna, P.J., Stip, E., Williams, R., MacEwan, G.W., Wasan, K., Procyshyn, R., 2006. Clozapine Alone versus Clozapine and Risperidone with Refractory Schizophrenia. N. Engl. J. Med. 354, 472–482. doi:10.1056/NEJMoa053222

Hong, C.J., Chen, J.Y., Chiu, H.J., Sim, C.B., 1997. A double-blind comparative study of clozapine versus chlorpromazine on Chinese patients with treatment-refractory schizophrenia. Int. Clin. Psychopharmacol. 12, 123–130.

Jayathilake, K., Meltzer, D., Small, J., Meltzer, H.Y., 2005. Comparison of clozapine and high dose olanzapine in treatment resistant schizophrenia in a double blind, randomized, 6 month clinical trial. Neuropsychopharmacology 30, S202.

Jenner, J.A., Nienhuis, F.J., van de Willige, G., Wiersma, D., 2006. “Hitting” voices of schizophrenia patients may lastingly reduce persistent auditory hallucinations and their burden: 18-month outcome of a randomized controlled trial. Can. J. Psychiatry Rev. Can. Psychiatr. 51, 169–177.

JIANG, X., YANG, K., ZHOU, B., JIN, P., 2009. Study on efficacy of modified electroconvulsive therapy(MECT)together with risperidone in treatment treatment-resistant schizophrenia(TRS). Chin. J. Nerv. Ment. Disord. 35, 79–83.

Josiassen, R.C., Joseph, A., Kohegyi, E., Stokes, S., Dadvand, M., Paing, W.W., Shaughnessy, R.A., 2005. Clozapine augmented with risperidone in the treatment of schizophrenia: a randomized, double-blind, placebo-controlled trial. Am. J. Psychiatry 162, 130–136. doi:10.1176/appi.ajp.162.1.130

Kane, J., Honigfeld, G., Singer, J., Meltzer, H., 1988. Clozapine for the treatment-resistant schizophrenic. A double-blind comparison with chlorpromazine. Arch. Gen. Psychiatry 45, 789–796.

Kane, J.M., Khanna, S., Rajadhyaksha, S., Giller, E., 2006. Efficacy and tolerability of ziprasidone in patients with treatment-resistant schizophrenia. Int. Clin. Psychopharmacol. 21, 21–28.

Kane, J.M., Kinon, B., Johns, C., 1993. Alternative strategies for treating neuroleptic non responsive patients. Schizophr. Res. 9, 240.

Kane, J.M., Marder, S.R., Schooler, N.R., Wirshing, W.C., Umbricht, D., Baker, R.W., Wirshing, D.A., Safferman, A., Ganguli, R., McMeniman, M., Borenstein, M., 2001. Clozapine and haloperidol in moderately refractory schizophrenia: a 6-month randomized and double-blind comparison. Arch. Gen. Psychiatry 58, 965–972.

Kane, J.M., Meltzer, H.Y., Carson, W.H., Jr, McQuade, R.D., Marcus, R.N., Sanchez, R., Aripiprazole Study Group, 2007. Aripiprazole for treatment-resistant schizophrenia: results of a multicenter, randomized, double-blind, comparison study versus perphenazine. J. Clin. Psychiatry 68, 213–223.

Kane, J.M., Potkin, S.G., Daniel, D.G., Buckley, P.F., 2011. A double-blind, randomized study comparing the efficacy and safety of sertindole and risperidone in patients with treatment-resistant schizophrenia. J. Clin. Psychiatry 72, 194–204. doi:10.4088/JCP.07m03733yel

Kenny, J., Meltzer, H., 1992. Effect of atypical and typical antipsychotic drugs on neuropsychological functions in early- stage schizophrenic patients, in: 7th Biennial Winter Workshop on Schizophrenia. Les Diablerets, Switzerland, p. 162.

Kim, J.M., Woo, H.W., 1997. The Therapeutic Effect of Sertraline in Treatment-resistant Schizophrenics. Korean J. Psychopharmacol. 8, 69–78.

Klírová, M., Horáček, J., Čermák, J., Novák, T., Tilerová, B., 2009. P02-83 Clinical response of neuronavigated rTMS in the treatment of auditory hallucinations. Eur. Psychiatry 24, S773–S773. doi:10.1016/S0924-9338(09)71006-5

Konrad, C., Schormair, C., Knickelbein, U., Ophaus, P., Eikelmann, B., 1997. Risperidone and clozapine in pharmaco-resistant schizophrenia. Pharmacopsychiatry 30, 190.

Kotler, M., Strous, R.D., Reznik, I., Shwartz, S., Weizman, A., Spivak, B., 2004. Sulpiride augmentation of olanzapine in the management of treatment-resistant chronic schizophrenia: evidence for improvement of mood symptomatology. Int. Clin. Psychopharmacol. 19, 23–26.

Kreinin, A., Novitski, D., Weizman, A., 2006. Amisulpride treatment of clozapine-induced hypersalivation in schizophrenia patients: a randomized, double-blind, placebo-controlled cross-over study. Int. Clin. Psychopharmacol. 21, 99–103.

Kremer, I., Vass, A., Gorelik, I., Bar, G., Blanaru, M., Javitt, D.C., Heresco-Levy, U., 2004. Placebo-controlled trial of lamotrigine added to conventional and atypical antipsychotics in schizophrenia. Biol. Psychiatry 56, 441–446. doi:10.1016/j.biopsych.2004.06.029

Krivoy, A., 2011. NCT01448499 Clozapine Versus Amisulpride in Treatment-resistant Schizophrenia Patients [WWW Document]. URL http://clinicaltrials.gov/ct2/show/NCT01448499 (accessed 3.28.14).

Kumra, S., Frazier, J.A., Jacobsen, L.K., McKenna, K., Gordon, C.T., Lenane, M.C., Hamburger, S.D., Smith, A.K., Albus, K.E., Alaghband-Rad, J., Rapoport, J.L., 1996. Childhood-onset schizophrenia. A double-blind clozapine-haloperidol comparison. Arch. Gen. Psychiatry 53, 1090–1097.

Kumra, S., Kranzler, H., Gerbino-Rosen, G., Kester, H.M., DeThomas, C., Cullen, K., Regan, J., Kane, J.M., 2008. Clozapine versus “high-dose” olanzapine in refractory early-onset schizophrenia: an open-label extension study. J. Child Adolesc. Psychopharmacol. 18, 307–316. doi:10.1089/cap.2007.0089

Kuwilsky, A., Krumm, B., Englisch, S., Dressing, H., Zink, M., 2010. Long-Term Efficacy and Tolerability of Clozapine Combined with Ziprasidone or Risperidone. Pharmacopsychiatry 43, 216–220. doi:10.1055/s-0030-1254089

Lal, S., Thavundayil, J.X., Nair, N.P.V., Annable, L., Ng Ying Kin, N.M.K., Gabriel, A., Schwartz, G., 2006. Levomepromazine versus chlorpromazine in treatment-resistant schizophrenia: a double-blind randomized trial. J. Psychiatry Neurosci. JPN 31, 271–279.

Lane, H.-Y., 2011. NCT01390376 DAAOI-1 Treatment for Treatment-resistant Schizophrenia - Full Text View - ClinicalTrials.gov [WWW Document]. URL http://clinicaltrials.gov/ct2/show/NCT01390376 (accessed 3.25.14).

Lee, M., Jung, I., Kwak, D., 1995. Clinical efficacy of clozapine in treatment-refractory schizophrenic patients, in: 8th Congress of the European College of Neuropsychopharmacology. Venice, Italy.

Lee, M.A., Thompson, P.A., Meltzer, H.Y., 1994. Effects of clozapine on cognitive function in schizophrenia. J. Clin. Psychiatry 55 Suppl B, 82–87.

Lee, S.-H., Kim, W., Chung, Y.-C., Jung, K.-H., Bahk, W.-M., Jun, T.-Y., Kim, K.-S., George, M.S., Chae, J.-H., 2005. A double blind study showing that two weeks of daily repetitive TMS over the left or right temporoparietal cortex reduces symptoms in patients with schizophrenia who are having treatment-refractory auditory hallucinations. Neurosci. Lett. 376, 177–181. doi:10.1016/j.neulet.2004.11.048

Lewis, S.W., Davies, L., Jones, P.B., Barnes, T.R.E., Murray, R.M., Kerwin, R., Taylor, D., Hayhurst, K.P., Markwick, A., Lloyd, H., Dunn, G., 2006. Randomised controlled trials of conventional antipsychotic versus new atypical drugs, and new atypical drugs versus clozapine, in people with schizophrenia responding poorly to, or intolerant of, current drug treatment. Health Technol. Assess. Winch. Engl. 10, iii–iv, ix–xi, 1–165.

Liberman, R.P., Gutkind, D., Mintz, J., Green, M., Marshall, B.D., Jr, Robertson, M.J., Hayden, J., 2002. Impact of risperidone versus haloperidol on activities of daily living in the treatment of refractory schizophrenia. Compr. Psychiatry 43, 469–473. doi:10.1053/comp.2002.33499

Lieberman, J., 2001. Risperidone and clozapine in chronic schizophrenia.

Lieberman, J., 2009. Tamoxifen for schizophrenia.

Lindenmayer, J.-P., Citrome, L., Khan, A., Kaushik, S., Kaushik, S., 2011. A randomized, double-blind, parallel-group, fixed-dose, clinical trial of quetiapine at 600 versus 1200 mg/d for patients with treatment-resistant schizophrenia or schizoaffective disorder. J. Clin. Psychopharmacol. 31, 160–168. doi:10.1097/JCP.0b013e31820f4fe0

Lindenmayer, J.-P., Park, M., Iskander, A., Bark, N., Smith, R., Cooper, T.B., 1996. Clozapine versus risperidone in treatment refractory state psychiatric inpatients, in: 149th Annual Meeting of the American Psychiatric Association. New York, USA, p. 231.

Louwerens, J., vdMeij, A., Slooff, C.J., 2000. Therapy resistance: the effectiveness of the second antipsychotic drug, a multicentre double blind comparative study (’switch study’). Schizophr. Res. 41, 183.

Lu, M.-L., Lane, H.-Y., Lin, S.-K., Chen, K.-P., Chang, W.-H., 2004. Adjunctive fluvoxamine inhibits clozapine-related weight gain and metabolic disturbances. J. Clin. Psychiatry 65, 766–771.

Ma, Q., Lian, H., Lili, X., 2007. A study of aripiprazole combined with clozapine in refractory schizophrenia. Med. J. Chin. Peoples Health 19, 93–99.

Marco, E.J., Wolkowitz, O.M., Vinogradov, S., Poole, J.H., Lichtmacher, J., Reus, V.I., 2002. Double-blind antiglucocorticoid treatment in schizophrenia and schizoaffective disorder: a pilot study. World J. Biol. Psychiatry Off. J. World Fed. Soc. Biol. Psychiatry 3, 156–161.

McCreadie, R.G., Flanagan, W.L., McKnight, J., Jorgensen, A., 1979. High dose flupenthixol decanoate in chronic schizophrenia. Br. J. Psychiatry J. Ment. Sci. 135, 175–179.

McCreadie, R.G., MacDonald, I.M., 1977. High dosage haloperidol in chronic schizophrenia. Br. J. Psychiatry J. Ment. Sci. 131, 310–316.

McEvoy, M.D., Joseph, for the CATIE Investigators, Lieberman, M.D., Jeffrey, Stroup, M.D., M.P.H. ,T., Davis, D.P.H., Sonia, Meltzer, M.D., Herbert, Rosenheck, M.D., Robert, Swartz, M.D., Marvin, Perkins, M.D., M.P.H. ,Diana, Keefe, P.D., Richard, Davis, P.D., Clarence, Severe, M.S., Joanne, Hsiao, M.D., John, 2006. Effectiveness of Clozapine Versus Olanzapine, Quetiapine, and Risperidone in Patients With Chronic Schizophrenia Who Did Not Respond to Prior Atypical Antipsychotic Treatment. Am. J. Psychiatry 163, 600–610. doi:10.1176/appi.ajp.163.4.600

McGurk, S.R., Carter, C., Goldman, R., Green, M.F., Marder, S.R., Xie, H., Schooler, N.R., Kane, J.M., 2005. The Effects of Clozapine and Risperidone on Spatial Working Memory in Schizophrenia. Am. J. Psychiatry 162, 1013–1016. doi:10.1176/appi.ajp.162.5.1013

McIntosh, A.M., Semple, D., Tasker, K., Harrison, L.K., Owens, D.G.C., Johnstone, E.C., Ebmeier, K.P., 2004. Transcranial magnetic stimulation for auditory hallucinations in schizophrenia. Psychiatry Res. 127, 9–17. doi:10.1016/j.psychres.2004.03.005

Meltzer, H., 2012. NCT01569659 High Dose Lurasidone for Patients With Treatment Resistant Schizophrenia [WWW Document]. URL http://clinicaltrials.gov/ct2/show/NCT01569659 (accessed 3.28.14).

Meltzer, H.Y., Bobo, W.V., Roy, A., Jayathilake, K., Chen, Y., Ertugrul, A., Anil Yağcioğlu, A.E., Small, J.G., 2008. A randomized, double-blind comparison of clozapine and high-dose olanzapine in treatment-resistant patients with schizophrenia. J. Clin. Psychiatry 69, 274–285.

Mercer, G., Finlayson, A., Johnstone, E.C., Murray, C., Owens, D.G., 1997. A study of enhanced management in patients with treatment-resistant schizophrenia. J. Psychopharmacol. Oxf. Engl. 11, 349–356.

Meskanen, K., Ekelund, H., Laitinen, J., Neuvonen, P.J., Haukka, J., Panula, P., Ekelund, J., 2013. A randomized clinical trial of histamine 2 receptor antagonism in treatment-resistant schizophrenia. J. Clin. Psychopharmacol. 33, 472–478. doi:10.1097/JCP.0b013e3182970490

Meszaros, K., Liechtenstein, A., Thau, K., Topitz, A., Simhandl, C., 1991. Lithium carbonate added to neuroleptics in treatment refractory schizophrenia. Schizophr. Res. - SCHIZOPHR RES 4, 291–2.

Meszaros, K., Simhandl, C., 1990. Carbamazepine as an adjunct of neuroleptics in chronic treatment refractory schizophrenia, in: 17th Collegium Internationale Neuro-Psychopharmacologicum Congress. Kyoto, Japan.

Mico’, U., Bruno, A., Pandolfo, G., Maria Romeo, V., Mallamace, D., D’Arrigo, C., Spina, E., Zoccali, R.A., Muscatello, M.R.A., 2011. Duloxetine as adjunctive treatment to clozapine in patients with schizophrenia: a randomized, placebo-controlled trial. Int. Clin. Psychopharmacol. 26, 303–310. doi:10.1097/YIC.0b013e32834bbc0d

Miyaoka, T., Furuya, M., Yasuda, H., Hayashida, M., Nishida, A., Inagaki, T., Horiguchi, J., 2009. Yi-gan san as adjunctive therapy for treatment-resistant schizophrenia: an open-label study. Clin. Neuropharmacol. 32, 6–9. doi:10.1097/WNF.0b013e31817e08c3

Mossaheb, N., Sacher, J., Wiesegger, G., 2006. Haloperidol in combination with clozapine in treatment-refractory patients with schizophrenia. J. Eur. Coll. Neuropsychopharmacol. 16, S416.

Muscatello, M.R.A., Bruno, A., Pandolfo, G., Micò, U., Bellinghieri, P.M., Scimeca, G., Cacciola, M., Campolo, D., Settineri, S., Zoccali, R., 2011. Topiramate augmentation of clozapine in schizophrenia: a double-blind, placebo-controlled study. J. Psychopharmacol. Oxf. Engl. 25, 667–674. doi:10.1177/0269881110372548

Muscatello, M.R.A., Bruno, A., Pandolfo, G., Micò, U., Scimeca, G., Di Nardo, F., Santoro, V., Spina, E., Zoccali, R.A., 2011. Effect of aripiprazole augmentation of clozapine in schizophrenia: a double-blind, placebo-controlled study. Schizophr. Res. 127, 93–99. doi:10.1016/j.schres.2010.12.011

Nair, C., Abraham, G., Stanilla, J.K., Simpson, G.M., Josiassen, R.C., 1997. Tardive dyskinesia and extrapyramidal symptoms in treatment-resistant schizophrenics treated with clozapine. Schizophr. Res. 24, 272–272. doi:10.1016/S0920-9964(97)82783-1

Neil A Rector, M.V.S., 2003. Cognitive therapy for schizophrenia: a preliminary randomized controlled trial. Schizophr. Res. 63, 1–11. doi:10.1016/S0920-9964(02)00308-0

Neppe, V.M., 1983. Non-responsive psychosis--a biochemical difference? South Afr. Med. J. Suid-Afr. Tydskr. Vir Geneeskd. 63, 797–798.

Neumann, R., 1988. ECT mit und ohne Neuroleptika bei Erkrankungen aus dem schizophrenen Formenkreis. Neuropsychiatrie 2, 293–8.

Nielsen, J., Emborg, C., Gydesen, S., Dybbro, J., Aagaard, J., Haderup, K., Glyngdal, P., Fabricius, S., Thode,, D., Lublin, H., Andersen, T., Damkier, P., Taylor, D., 2012. Augmenting Clozapine With Sertindole: A Double-Blind, Randomized, Placebo-Controlled Study. J. Clin. Psychopharmacol. 32, 173–178. doi:10.1097/JCP.0b013e318248dfb8

Ojeda, N., Peña, J., Sánchez, P., Bengoetxea, E., Elizagárate, E., Ezcurra, J., Gutiérrez Fraile, M., 2012. Efficiency of cognitive rehabilitation with REHACOP in chronic treatment resistant Hispanic patients. NeuroRehabilitation 30, 65–74. doi:10.3233/NRE-2011-0728

Oleneva, E., 2005. Combined olanzapine-ECT therapy for resistant schizophrenia. Eur. Neuropsychopharmacol. 15, S131.

Owen, M., 1996. Antiglucocorticoids in depression and schizophrenia, in: 149th Annual Meeting of the American Psychiatric Association. New York, USA.

Owens, D.G., Harrison-Read, P.E., Johnstone, E.C., 1994. L-dopa helps positive but not negative features of neuroleptic-insensitive chronic schizophrenia. J. Psychopharmacol. Oxf. Engl. 8, 204–212. doi:10.1177/026988119400800403

Palm, U., 2011. NCT01378078 Transcranial Direct Current Stimulation (tDCS) for Treatment of Negative Syndrome in Patients With Schizophrenia [WWW Document]. URL http://clinicaltrials.gov/ct2/show/ (accessed 3.18.14).

Peng, H., Kuang, Y., Huang, X., 2001. A Control Study of Risperidone in Combination with Clozapine in Treating Refractory Schizophrenia. J. Mod. Clin. Med. Bioeng. 7, 100–2.

Penn, D., 2004. A 24-week investigation of group cognitive behavioral therapy (CBT) for medication-resistant auditory hallucinations in 60 patients. Stanley Found. Res. Programs.

Pickar, D., Owen, R.R., Litman, R.E., Konicki, E., Gutierrez, R., Rapaport, M.H., 1992. Clinical and biologic response to clozapine in patients with schizophrenia. Crossover comparison with fluphenazine. Arch. Gen. Psychiatry 49, 345–353.

Pinto, A., La Pia, S., Mennella, R., Giorgio, D., DeSimone, L., 1999. Cognitive-behavioral therapy and clozapine for clients with treatment-refractory schizophrenia. Psychiatr. Serv. Wash. DC 50, 901–904.

Potkin, S.G., Basile, V.S., Jin, Y., Masellis, M., Badri, F., Keator, D., Wu, J.C., Alva, G., Carreon, D.T., Bunney, W.E., Jr, Fallon, J.H., Kennedy, J.L., 2003. D1 receptor alleles predict PET metabolic correlates of clinical response to clozapine. Mol. Psychiatry 8, 109–113. doi:10.1038/sj.mp.4001191

Potkin, S.G., Bera, R., Gulasekaram, B., Costa, J., Hayes, S., Jin, Y., Richmond, G., Carreon, D., Sitanggan, K., Gerber, B., 1994. Plasma clozapine concentrations predict clinical response in treatment-resistant schizophrenia. J. Clin. Psychiatry 55 Suppl B, 133–136.

Potkin, S.G., Jin, Y., Bunney, B.G., Costa, J., Gulasekaram, B., 1999. Effect of clozapine and adjunctive high-dose glycine in treatment-resistant schizophrenia. Am. J. Psychiatry 156, 145–147.

Quitkin, F., Rifkin, A., Klein, D.F., 1975. Very high dosage vs standard dosage fluphenazine in schizophrenia. A double-blind study of nonchronic treatment-refractory patients. Arch. Gen. Psychiatry 32, 1276–1281.

Remington, G., Kapur, S., Foussias, G., Agid, O., Mann, S., Borlido, C., Richards, S., Javaid, N., 2012. Tetrabenazine augmentation in treatment-resistant schizophrenia: a 12-week, double-blind, placebo-controlled trial. J. Clin. Psychopharmacol. 32, 95–99. doi:10.1097/JCP.0b013e31823f913e

Richardson CM, Feldman S, Kelly DL, 2009. Metabolic side effects of combined antipsychotic treatment: results from a double blind trial of adjunctive risperidone in clozapine treated people with treatment-resistant schizophrenia. Schizophr. Bull. 35, 38–9.

Rosa, M., Gattaz, W., Rosa, M., Rumi, D., Tavares, H., Myczkowski, H., Sartorelli, M., Rigonatti, S., Elkis, H., Cabral, S., Teixeira, M., Marcolin, M., 2007. Effects of repetitive transcranial magnetic stimulation on auditory hallucinations refractory to clozapine. J. Clin. Psychiatry 68, 1528–1532.

Rosenheck, R., Charney, D.S., Frisman, L.K., Cramer, J., 1995. Clozapine’s cost effectiveness. Am. J. Psychiatry 152, 152–153.

S. Grenier, G.F., 2008. 55 – Repetitive transcranial magnetic stimulation efficiency in treatment-resistant auditory hallucinations. Schizophr. Res. - SCHIZOPHR RES 98, 56–56. doi:10.1016/j.schres.2007.12.122

Sacchetti, E., Galluzzo, A., Valsecchi, P., Romeo, F., Gorini, B., Warrington, L., MOZART Study Group, 2009. Ziprasidone vs clozapine in schizophrenia patients refractory to multiple antipsychotic treatments: the MOZART study. Schizophr. Res. 113, 112–121.

Schulman, A., Wetterberg, L., Asaba, H., 1983. [Hemodialysis of therapy-resistant patients with chronic schizophrenia]. Läkartidningen 80, 2641–2642.

Schulz, S.C., Thompson, P.A., Jacobs, M., Ninan, P.T., Robinson, D., Weiden, P.J., Yadalam, K., Glick, I.D., Odbert, C.L., 1999. Lithium augmentation fails to reduce symptoms in poorly responsive schizophrenic outpatients. J. Clin. Psychiatry 60, 366–372.

Sensky, T., Turkington, D., Kingdon, D., Scott, J.L., Scott, J., Siddle, R., O’Carroll, M., Barnes, T.R., 2000. A randomized controlled trial of cognitive-behavioral therapy for persistent symptoms in schizophrenia resistant to medication. Arch. Gen. Psychiatry 57, 165–172.

Shaw, P., Sporn, A., Gogtay, N., Overman, G.P., Greenstein, D., Gochman, P., Tossell, J.W., Lenane, M., Rapoport, J.L., 2006. Childhood-onset schizophrenia: A double-blind, randomized clozapine-olanzapine comparison. Arch. Gen. Psychiatry 63, 721–730. doi:10.1001/archpsyc.63.7.721

Shekhar, A., 2005. NCT00216281 Efficacy and Safety Study of Clozapine Augmented by Atomoxetine Versus Clozapine Augmented by Placebo in Patients With Chronic Resistant Schizophrenia [WWW Document]. URL http://clinicaltrials.gov/ct2/show/NCT00216281 (accessed 3.18.14).

Shiloh, R., Zemishlany, Z., Aizenberg, D., Valevski, A., Bodinger, L., Munitz, H., Weizman, A., 2002. Mianserin or placebo as adjuncts to typical antipsychotics in resistant schizophrenia. Int. Clin. Psychopharmacol. 17, 59–64.

Silverstein, S.M., Spaulding, W.D., Menditto, A.A., Savitz, A., Liberman, R.P., Berten, S., Starobin, H., 2009. Attention Shaping: a Reward-Based Learning Method to Enhance Skills Training Outcomes in Schizophrenia. Schizophr. Bull. 35, 222–232. doi:10.1093/schbul/sbm150

Simhandl, C., Meszaros, K., Denk, E., Thau, K., Topitz, A., 1996. Adjunctive carbamazepine or lithium carbonate in therapy-resistant chronic schizophrenia. Can. J. Psychiatry Rev. Can. Psychiatr. 41, 317.

Simpson, G.M., Josiassen, R.C., Stanilla, J.K., de Leon, J., Nair, C., Abraham, G., Odom-White, A., Turner, R.M., 1999. Double-blind study of clozapine dose response in chronic schizophrenia. Am. J. Psychiatry 156, 1744–1750.

Slotema, C.W., Blom, J.D., de Weijer, A.D., Diederen, K.M., Goekoop, R., Looijestijn, J., Daalman, K., Rijkaart, A.-M., Kahn, R.S., Hoek, H.W., Sommer, I.E.C., 2011. Can low-frequency repetitive transcranial magnetic stimulation really relieve medication-resistant auditory verbal hallucinations? Negative results from a large randomized controlled trial. Biol. Psychiatry 69, 450–456. doi:10.1016/j.biopsych.2010.09.051

Slotema, C.W., Blom, J.D., de Weijer, A.D., Hoek, H.W., Sommer, I.E., 2012. Priming does not enhance the efficacy of 1 Hertz repetitive transcranial magnetic stimulation for the treatment of auditory verbal hallucinations: results of a randomized controlled study. Brain Stimulat. 5, 554–559. doi:10.1016/j.brs.2011.10.005

Small, J.G., Klapper, M.H., Malloy, F.W., Steadman, T.M., 2003. Tolerability and efficacy of clozapine combined with lithium in schizophrenia and schizoaffective disorder. J. Clin. Psychopharmacol. 23, 223–228. doi:10.1097/01.jcp.0000084026.22282.5f

Smith, R.C., Infante, M., Singh, A., Khandat, A., 2001. The effects of olanzapine on neurocognitive functioning in medication-refractory schizophrenia. Int. J. Neuropsychopharmacol. Off. Sci. J. Coll. Int. Neuropsychopharmacol. CINP 4, 239–250.

Tamminga, C.A., Schaffer, M.H., Smith, R.C., Davis, J.M., 1978. Schizophrenic symptoms improve with apomorphine. Science 200, 567–568.

Tang, W.-K., Ungvari, G.S., 2003. Efficacy of electroconvulsive therapy in treatment-resistant schizophrenia: a prospective open trial. Prog. Neuropsychopharmacol. Biol. Psychiatry 27, 373–379. doi:10.1016/S0278-5846(02)00354-8

Tarrier, N., Beckett, R., Harwood, S., Baker, A., Yusupoff, L., Ugarteburu, I., 1993. A trial of two cognitive-behavioural methods of treating drug-resistant residual psychotic symptoms in schizophrenic patients: I. Outcome. Br. J. Psychiatry J. Ment. Sci. 162, 524–532.

Tiihonen, J., Hallikainen, T., Ryynänen, O.-P., Repo-Tiihonen, E., Kotilainen, I., Eronen, M., Toivonen, P., Wahlbeck, K., Putkonen, A., 2003. Lamotrigine in treatment-resistant schizophrenia: a randomized placebo-controlled crossover trial. Biol. Psychiatry 54, 1241–1248.

Tiihonen, J, Halonen P, Wahlbeck, K, Repo-Tiihonen, E, Hyvärinen S, Eronen, M, Putkonen, H, Takala P, Mehtonen, Op, Puck, M, J, O., P, K., G, J., J, A., T, H., Op, R., E, T., 2005. Topiramate add-on in treatment-resistant schizophrenia: a randomized, double-blind, placebo-controlled, crossover trial. J. Clin. Psychiatry 66, 1012–1015.

Tollefson, G.D., Birkett, M.A., Kiesler, G.M., Wood, A.J., Lilly Resistant Schizophrenia Study Group, 2001. Double-blind comparison of olanzapine versus clozapine in schizophrenic patients clinically eligible for treatment with clozapine. Biol. Psychiatry 49, 52–63.

Valmaggia, L.R., Gaag, M.V.D., Tarrier, N., Pijnenborg, M., Slooff, C.J., 2005. Cognitive–behavioural therapy for refractory psychotic symptoms of schizophrenia resistant to atypical antipsychotic medication Randomised controlled trial. Br. J. Psychiatry 186, 324–330. doi:10.1192/bjp.186.4.324

VanderZwaag, C., McGee, M., McEvoy, J.P., Freudenreich, O., Wilson, W.H., Cooper, T.B., 1996. Response of patients with treatment-refractory schizophrenia to clozapine within three serum level ranges. Am. J. Psychiatry 153, 1579–1584.

Vercammen, A., Knegtering, H., Bruggeman, R., Westenbroek, H.M., Jenner, J.A., Slooff, C.J., Wunderink, L., Aleman, A., 2009. Effects of bilateral repetitive transcranial magnetic stimulation on treatment resistant auditory-verbal hallucinations in schizophrenia: a randomized controlled trial. Schizophr. Res. 114, 172–179. doi:10.1016/j.schres.2009.07.013

Vercammen, A., Knegtering, H., Liemburg, E.J., den Boer, J.A., Aleman, A., 2010. Functional connectivity of the temporo-parietal region in schizophrenia: effects of rTMS treatment of auditory hallucinations. J. Psychiatr. Res. 44, 725–731. doi:10.1016/j.jpsychires.2009.12.011

Vital-Herne, J., Gerbino, L., Kay, S.R., Katz, I.R., Opler, L.A., 1986. Mesoridazine and thioridazine: clinical effects and blood levels in refractory schizophrenics. J. Clin. Psychiatry 47, 375–379.

Volavka, J., Czobor, P., Sheitman, B., Lindenmayer, J.-P., Citrome, L., McEvoy, J.P., Cooper, T.B., Chakos, M., Lieberman, J.A., 2002. Clozapine, olanzapine, risperidone, and haloperidol in the treatment of patients with chronic schizophrenia and schizoaffective disorder. Am. J. Psychiatry 159, 255–262.

Wahlbeck, K., Cheine, M., Tuisku, K., Ahokas, A., Joffe, G., Rimón, R., 2000. Risperidone versus clozapine in treatment-resistant schizophrenia: a randomized pilot study. Prog. Neuropsychopharmacol. Biol. Psychiatry 24, 911–922.

Wang, B., 2008. Efficacy of modified electroconvulsive therapy in trea tment refractory schizophrenia. Linchuang Jingshen Yixue Zazhi 18, 415–7.

Wang, X., Shong, Y., Xia, Y., Wang, L., 2003. ILLLI Treatment on the Intractable Schizophrenia．A Control Study. Chin. J. Laser Med. Surg. 12, 114–8.

Weiner, E., Conley, R.R., Ball, M.P., Feldman, S., Gold, J.M., Kelly, D.L., Wonodi, I., McMahon, R.P., Buchanan, R.W., 2010. Adjunctive risperidone for partially responsive people with schizophrenia treated with clozapine. Neuropsychopharmacol. Off. Publ. Am. Coll. Neuropsychopharmacol. 35, 2274–2283. doi:10.1038/npp.2010.101

Wilson, W.H., 1993. Addition of lithium to haloperidol in non-affective, antipsychotic non-responsive schizophrenia: a double blind, placebo controlled, parallel design clinical trial. Psychopharmacology (Berl.) 111, 359–366.

Xiong, D., Liu, L., Yi-Yan, Ye-Feng, 2010. [Observation on the therapeutic effect of electroacupuncture combined with small dose of clozapine in clinical treatment of refractory schizophrenia]. Zhen Ci Yan Jiu Acupunct. Res. Zhongguo Yi Xue Ke Xue Yuan Yi Xue Qing Bao Yan Jiu Suo Bian Ji 35, 134–137.

Zastowny, T.R., Lehman, A.F., Cole, R.E., Kane, C., 1992. Family management of schizophrenia: A comparison of behavioral and supportive family treatment. Psychiatr. Q. 63, 159–186. doi:10.1007/BF01065988

Zhang, X.Y., Zhou, D.F., Cao, L.Y., Zhang, P.Y., Wu, G.Y., Shen, Y.C., 2001a. Risperidone versus haloperidol in the treatment of acute exacerbations of chronic inpatients with schizophrenia: a randomized double-blind study. Int. Clin. Psychopharmacol. 16, 325–330.

Zhang, X.Y., Zhou, D.F., Zhang, P.Y., Wu, G.Y., Su, J.M., Cao, L.Y., 2001b. A double-blind, placebo-controlled trial of extract of Ginkgo biloba added to haloperidol in treatment-resistant patients with schizophrenia. J. Clin. Psychiatry 62, 878–883.

Zhang, Z.-J., Kang, W.-H., Li, Q., Wang, X.-Y., Yao, S.-M., Ma, A.-Q., 2006. Beneficial effects of ondansetron as an adjunct to haloperidol for chronic, treatment-resistant schizophrenia: a double-blind, randomized, placebo-controlled study. Schizophr. Res. 88, 102–110. doi:10.1016/j.schres.2006.07.010

Zheng, 2006. Nursing intervention on family rehabilitation of chronic refractory schizophrenic patients. 中华护理月刊 93, 11–12.

Zhou, Y., Wang, Z., Zhang, W., 2004. Comparative analysis between bilateral brain stereotactic anterior destruction tactics and the schizophrenia treated with the medicine of resistant mental disease. 中华实用医学 6, 16–20.

Zhu, H., Deng, D., Yu, G., 2002. A study of clozapine combined with or without pipotiazine palmitate in refractory schizophrenia. J. Clin. Psychol. Med. 12, 15–7.

Zhu, Q., Pi, P., 2012. A comparative study of paliperidone and olanzapine in the treatment of treatment-refractory schizophrenia. Chin. J. New Drugs 21, 666–9.

Zoccali, R., Muscatello, M.R., Bruno, A., Cambria, R., Micò, U., Spina, E., Meduri, M., 2007. The effect of lamotrigine augmentation of clozapine in a sample of treatment-resistant schizophrenic patients: a double-blind, placebo-controlled study. Schizophr. Res. 93, 109–116. doi:10.1016/j.schres.2007.02.009

于彩霞, 冀德才, 袁航, 刘勇, 2006. 阿立哌唑治疗难治性精神分裂症的疗效观察. 山东精神医学 19, 263–265.

任列, 朱毅平, 孙菊水, 2009. 齐拉西酮联合小剂量氯氮平治疗难治性精神分裂症30例. 医药导报 28, 872–874.

任进军, 苏俊玲, 2000. 利培酮和氯氮平治疗难治性精神分裂症对比研究. 包头医学 24, 5–6.

何燕飞, 谢汪传, 李志云, 2010. 利培酮合用碳酸锂治疗难治性精神分裂症的对照研究. 广州医药 41, 6–8.

余国汉, 黄雄, 1999. 利培酮和氯氮平治疗难治性精神分裂症对照观察. 中国神经精神疾病杂志 25, 366–367.

冯春霞, 黄世勋, 杨洪志, 陈九义, 刘和祥, 2001. 利培酮与氯丙嗪治疗难治性精神分裂症对照研究. 山东精神医学 14, 95–96.

冯雪雁, 彭华, 2002. 维思通与氯氮平合并治疗难治性精神分裂症的临床分析. Int. Chin. Neuropsychiatry Med. J. 3, 109–10.

刘伟锋, 张卫敏, 2008. 阿立哌唑与利培酮治疗难治性精神分裂症疗效观察. 中国民康医学.

刘佰聪, 2007. 奎硫平治疗难治性精神分裂症的疗效观察. 中国现代医生 45, 24–25.

刘友夺, 黄文芳, 黄时金, 2008. 氯氮平合癸氟奋乃静治疗难治性精神 分裂症对照研究. Med. J. Chin. People′s Health 20, 1274.

刘发强, 龚高钦, 2010. 精神分裂症患者无抽搐电休克的效果. 中国现代医生 48, 29, 36.

刘春仙, 刘胜皇, 涂哲明, 刘波, 2005. 奎硫平合并小剂量氯氮平治疗难治性精神分裂症的临床对照研究. 中国民康医学 17, 748–749.

刘绍梅, 谷瑞莲, 肖培贤, 2001. 利培酮和氟奋乃静治疗难治性精神分裂症的对照研究. 山东精神医学 14, 202–204.

刘英, 2005. 维思通与氯氮平治疗难治性精神分裂症的临床对照研究. 菏泽医学专科学校学报 17, 13–14.

吴丽会, 2002. 利培酮合并氯氮平治疗难治性精神分裂症的对照研究. 健康心理学杂志 10, 135–137.

吴树靖, 邢桂芳, 2002. 维思通和氯丙嗪治疗慢性精神分裂症的对照研究. 健康心理学杂志 10, 364–365.

唐振坤, 陈德彩, 孟宪礼, 2007. 奥氮平与氯氮平治疗难治性精神分裂症对照研究. 中国民康医学 19, 1021–1022.

唐步春, 陆志新, 周振和, 2005. 齐拉西酮与氯氮平治疗难治性精神分裂症对照研究. 临床心身疾病杂志 11, 303–304.

孔庆任, 杨瑞香, 李守春, 卢振胜, 李俊业, 陈霞, 2001. 氯氮平合并利培酮与氯氮平合并舒必利治疗难治性精神分裂症的对照研究. 山东精神医学 14, 119–120.

孙祺章, 梁景省, 余国汉, 黄雄, 2001. 利培酮与氯氮平治疗难治性精神分裂症阴性症状的对照研究. 齐齐哈尔医学院学报 22, 1247–1248.

孙辉, 焦玉涛, 张鲁新, 2008. 氯氮平合并碳酸锂治疗男性难治性精神分裂症的对照研究. 四川精神卫生 21, 83–85.

张仁凯, 付学凯, 王建利, 张春鹏, 2008. 阿立哌唑与氯氮平治疗难治性精神分裂症的对照研究. 齐齐哈尔医学院学报 29, 1703–1704.

张启林, 翟江, 2010. 阿立哌唑与氯氮平治疗难治性精神分裂症的对照研究. 华西医学 1033–1035.

张建新, 朱凤艳, 石夏明, 张喜梅, 魏立和, 吉中孚, 林瑞明, 2005. 利培酮和氯氮平治疗伴抑郁症状的难治性精神分裂症对照研究. 临床精神医学杂志 15, 201–202.

张星亮, 刘爱国, 高班玲, 2012. 阿立哌唑与氯氮平治疗难治性精神分裂症对照研究. 实用医技杂志 19, 1334–1335.

张艳琦, 于振东, 2009. 阿立哌唑与氯氮平治疗难治性精神分裂症疗效比较观察. 人民军医 52, 822–823.

张艳琦, 于振东, 2010. 齐拉西酮与氯氮平治疗难治性精神分裂症对照研究. 中国民康医学 22, 532–533.

张轶杰, 刘琼, 胡怡, 周利国, 2010. 无抽搐电痉挛治疗合并氯氮平治疗难治性 精神分裂症及其对记忆力的影响. Chin. Ment. Health J. 24, 440–4.

徐儒瑾, 万学东, 杜春秀, 吴乐平, 2008. 利培酮联合氯氮平治疗难治性精神分裂症. 中国现代药物应用 2, 78–78.

曹红军, 尤海峰, 范凤兰, 张静, 2003. 利培酮和氯氮平治疗难治性精神分裂症的对照研究. J. Chin. Medi Jne Res. 3, 316–9.

朱倩芸, 张绍文, 皮峻峰, 2003. 利培酮治疗难治性精神分裂症15例. 医药导报.

朱凤艳, 林瑞明, 张建新, 张喜梅, 甘明远, 吉中孚, 2003. 利培酮和氯氮平治疗难治性精神分裂症的随机对照研究. 上海精神医学 168–171.

朱华芳, 2007. 利培酮和氟派啶醇治疗难治性精神分裂症的对照研究. 中国保健 15, 42–43.

朱琛擘, 范勇, 王立钢, 孙艳, 2008. 阿立哌唑与氟哌啶醇治疗难治性精神分裂症的对照研究. 中外健康文摘：医药月刊 5, 3–4.

李晓菊, 罗捷, 段彪, 夏先容, 2009. 国产奥氮平与氯氮平治疗难治性精神分裂症的对照研究. 重庆医学 38, 783–784.

李爱凤, 姚建新, 廖小华, 劳永志, 蒋泽宇, 童梓顺, 2007. 国产阿立哌唑与氯氮平治疗难治性精神分裂症的对照研究. 中国民康医学 19, 165–167.

杨彦林, 2006. 奎硫平与氯氮平治疗难治性精神分裂症的对照研究. 实用心脑肺血管病杂志 14, 276–277.

杨甫德, 李尚明, 张兴理, 李娟, 田玉英, 姚付新, 陈大春, 2005. 利培酮和氯氮平治疗难治性精神分裂症的随机对照研究. 中国新药杂志 14, 610–613.

杨福收, 杨彦林, 张朝辉, 2004. 奎硫平与氯氮平治疗难治性精神分裂症对照研究. 中国民康医学 16, 12–13.

杨立身, 李正才, 吴英, 曾昭纬, 周晓云, 张嗣兴, 2004. 利培酮不同剂量治疗难治性精神分裂症对照研究. 临床心身疾病杂志 10, 158–160.

杨立身, 李正才, 王卫生, 曾昭伟, 2005. 奥氮平与氯氮平治疗难治性精神分裂症对照研究. 临床心身疾病杂志 11, 110–112.

林卓毅, 杨孝, 李福球, 苏伟胜, 2009. 齐拉西酮、氯氮平治疗的卫生经济学比较. 临床精神医学杂志 19, 97–98.

段武钢, 曾德志, 罗建武, 周桂明, 商秀珍, 罗世芳, 2010. 宁心汤对难治性精神分裂症治疗的增效作用. 临床精神医学杂志 20, 188–189.

毛星, 刘洪秋, 陈玉辉, 2010. 奥氮平与阿立哌唑治疗难治性精神分裂症70例的对照研究. 中国民康医学 22, 1365–1366.

潘朝霞, 尹定富, 刘宁汉, 2010. 丙戊酸钠联合氯氮平治疗难治性精神分裂症34例. 医药导报 203–204.

王刚平, 颉瑞, 裴根祥, 张学军, 2009. 丙戊酸镁缓释片治疗精神分裂症的辅助作用. 临床精神医学杂志 250–251.

王勇, 陈祖金, 袁洪彬, 2010. 奎硫平与利培酮治疗女性难治性精神分裂症对照研究. 中国民康医学 22, 2193–2195.

王强, 1995. 氯丙嗪合并碳酸锂治疗难治性精神分裂症随机双盲交叉试验. 临床精神医学杂志 5, 185.

王敬龙, 王建平, 2010. 喹硫平联合利培酮治疗难治性精神分裂症的疗效观察. 中国现代药物应用 127–128.

王淑芳, 邢君, 2010. 利培酮和氯氮平治疗难治性精神分裂症的临床对照分析. 中国民康医学 22, 2052–2054.

王秀梅, 李刚, 李忠义, 2010. 奎硫平与氯氮平治疗难治性精神分裂症的对照研究. 中国民康医学 22, 819–820.

王立钢, 刘延梅, 万好, 2004. 奥氮平与氯丙嗪治疗难治性精神分裂症的对照研究. 健康心理学杂志 12, 203–204.

王艳君, 2007. 奥氮平与氯氮平治疗难治性精神分裂症的对照研究. 实用药物与临床 10, 348–349.

王荣芝, 耿玉友, 潘德花, 张三晋, 2002. 利培酮与氯氮平治疗难治性精神分裂症对照研究. 山东精神医学 15, 221–222.

程绍忠, 迟秀芝, 李清华, 姜翕球, 2002. 利培酮与氯氮平治疗难治性精神分裂症对照研究. 健康心理学杂志 10, 44–46.

莫亚莉, 2010. 齐拉西酮联合小剂量氯氮平治疗难治性精神分裂症33例疗效观察. 浙江实用医学 43–45.

蒋国庆, 罗捷, 2009. 阿立哌唑与氯氮平治疗难治性精神分裂症的随机对照研究. 重庆医学 38, 87–88.

贡永宁, 郭平, 2010. 丙戊酸镁对难治性精神分裂症患者认知功能的影响. 慢性病学杂志 12, 107–109.

邓世平, 刘小花, 邹晓华, 潘大津, 徐哲明, 2006. 氯氮平联合肌苷口服液治疗难治性精神分裂症30例. 医药导报 25, 789–791.

邬德纯, 刘永忠, 罗来兴, 邬素萍, 2005. 奥氮平合并舒必利治疗难治性精神分裂症的临床对照研究. 中国行为医学科学 14, 639–641.

邹果果, 黄弋谨, 邹思义, 杨叶珍, 2003. 氯氮平合并舒必利治疗难治性精神分裂症临床对照研究. J. Yichun Univ. 25, 94–6.

郑占杰, 刘琳琳, 王骞, 2003. 利培酮与氟哌啶醇治疗难治性精神分裂症的对照研究. 山东精神医学 16, 220–221.

郑晖, 许崇涛, 2001. 氯氮平与奥氮平治疗难治性精神分裂症的临床观察. 齐齐哈尔医学院学报 22, 865–865.

郭建雄, 李婷, 麦桂英, 王西林, 杜文佳, 2003. 氯丙嗪合并低剂量的奥氮平治疗难治性精神分裂症的临床观察. 中国现代医学杂志 13, 49–50.

闫宝昌, 2008. 阿立哌唑与氯氮平治疗难治性精神分裂症的对照观察. 中国误诊学杂志 8, 4304–4305.

阮江红, 黄国红, 2005. 奎硫平与氯氮平治疗难治性精神分裂症对照研究. 中国民康医学 17, 747–748.

陈丽霞, 田常青, 2010. 利培酮治疗难治性精神分裂症随机对照研究. 疾病监测与控制杂志 102–104.

陈列, 张湘, 李健, 阎翰, 盛小奇, 谌益华, 2001. 氯氮平与碳酸锂联合治疗难治性精神分裂症的效果分析. 四川精神卫生 14, 213–214.

陈眷梅, 于东升, 赵红伟, 2003. 利培酮合并氯氮平治疗难治性精神分裂症. 中华临床医药杂志 68, 11274–5.

韩鹏, 2000. 利培酮及氯氮平治疗难治性精神分裂症疗效观察. 济宁医学院学报 23, 75–75.

马迅, 2010. 利培酮与氯氮平治疗难治性精神分裂症的临床对照研究. 中国民康医学 22, 2752–2753.

高军, 张伟, 2010. 奥氮平与氯氮平治疗难治性精神分裂症的对照研究. 实用医药杂志 27, 577–578.

高军, 张文兵, 2006. 利培酮与氯氮平治疗难治性精神分裂症对照研究. 临床心身疾病杂志 12, 346–347.

高桂林, 李翠茹, 2006. 奥氮平与氯氮平治疗难治性精神分裂症对照研究. 山东精神医学 19, 96–97.

黄祖荣, 林家幸, 余木英, 2009. 利培酮联合小剂量氯氮平治疗难治性精神分裂症的疗效研究. 河北医学 15, 133–136.
